# Identifying American climate change free riders and motivating sustainable behavior

**DOI:** 10.1038/s41598-024-57042-w

**Published:** 2024-03-19

**Authors:** Beatrice Magistro, Cecilia Abramson, Daniel Ebanks, Ramit Debnath, R. Michael Alvarez

**Affiliations:** 1 California Institute of Technology, Pasadena, CA 91125 USA; 2 Harvard University, Cambridge, MA 02138 USA; 3 University of Cambridge, Cambridge, CB30HE UK

**Keywords:** Collective action, Sustainability behaviors, Environment, Climate change, Free riders, Psychology and behaviour, Sustainability

## Abstract

Free riders, who benefit from collective efforts to mitigate climate change but do not actively contribute, play a key role in shaping behavioral climate action. Using a sample of 2096 registered American voters, we explore the discrepancy between two groups of free riders: cynics, who recognize the significance of environmental issues but do not adopt sustainable behaviors, and doubters, who neither recognize the significance nor engage in such actions. Through statistical analyses, we show these two groups are different. Doubters are predominantly male, younger, with lower income and education, exhibit stronger conspiracy beliefs, lower altruism, and limited environmental knowledge, are more likely to have voted for Trump and lean towards conservative ideology. Cynics are younger, religious, higher in socioeconomic status, environmentally informed, liberal-leaning, and less likely to support Trump. Our research provides insights on who could be most effectively persuaded to make climate-sensitive lifestyle changes and provides recommendations to prompt involvement in individual sustainability behaviors. Our findings suggest that for doubters, incentivizing sustainability through positive incentives, such as financial rewards, may be particularly effective. Conversely, for cynics, we argue that engaging them in more community-driven and social influence initiatives could effectively translate their passive beliefs into active participation.

## Introduction

Climate change mitigation requires action at multiple levels, from international and national policies to local community initiatives. However, at its core, successful mitigation also hinges upon individual behavioral changes, which may necessitate significant alterations to people’s lifestyles. Yet, mitigating climate change presents a collective action problem, as it demands the cooperation of all individuals. This challenge becomes apparent as we observe that a majority of people fail to engage in sustainable behaviors^1,2^. Understanding who these individuals are is crucial for identifying the most effective strategies to motivate climate-minded lifestyle changes.

This effort to motivate collective pro-environmental behavior is rooted in the evolving definitions of sustainable communities^3,4^. For example, Biancardi et al.^5^ present a comprehensive overview of sustainable energy communities and state that such communities strive for net-zero action with a broader contribution to the advancement of a green economy. There are specific social and structural arrangements needed to stabilize such communities influenced by family and social networks^6,7^. However, the authors caution that these factors are sensitive to local contexts and cultures, and there is a need to study the underlying drivers of sustainable behaviors.

Our research question is grounded in the literature focusing on collective action challenges and individual behaviors in such contexts. Olson’s influential work, “The Logic of Collective Action”, underscores the necessity of cooperation for attaining public goods^8^. Within sustainability, we define the public good as a “clean environment”, encompassing elements like air quality and biodiversity. Drawing on Olson’s collective action analysis, we acknowledge “free riders”-those benefiting from the public good without engaging in sustainability^8^. Specifically, we focus on 7 sustainable behaviors: reducing electricity use, minimizing meat consumption, driving less, using fewer plastics, reducing food waste, reducing water consumption, and composting practices. Our study aims to identify and analyze the characteristics associated with skepticism or inaction towards sustainable behaviors, specifically focusing on two distinct groups of free-riders: those who understand that human actions contribute to climate change and recognize the importance of shifting behaviors but do not actively engage in sustainability practices, who we call “cynics”, and those that neither recognize the importance of these actions nor they engage in them, who we call “doubters”. By understanding these characteristics, we hope to provide valuable recommendations and potential implications for the development of effective interventions that can ultimately induce positive behavioral change.

Demographically, the literature has identified a number of variables that predict whether people will or will not engage in sustainability behaviors. Political orientation, which includes both political ideology and party affiliation, stands as a key determinant of overall environmental attitudes and behaviors, with particularly pronounced effects in the U.S., whereby individuals who are politically conservative and Republican are less likely to adopt behaviors that help fight climate change^9–16^. Men in general are less likely to take action to reduce climate change and to engage in pro-environment behaviors, which is consistent with gender socialization theory, according to which women are typically raised to embrace a feminine identity that stresses cooperation, empathy, and care, as opposed to masculine identities stressing dominance, competition, and individualism^10,14,17–19^. Post-modernism predicts that environmental concerns should become more prominent as people satisfy their fundamental material needs and attain political and emotional stability^20^. Younger adults, who have grown up recently under wealthier circumstances, and individuals who enjoy a higher socioeconomic position, as indicated by their income and advanced degrees, should be more likely to express stronger pro-environmental views compared to their counterparts^20^, however the empirical support for this theory is rather mixed^10,21^.

The relationship between religious beliefs and environmental and climate concerns is complex^22^. Studies indicate a correlation between religious background and environmental attitudes, showing that religious individuals tend to be less environmentally conscious compared to their non-religious counterparts^23^. This trend is especially pronounced in the United States among Evangelical Protestants, Catholics, and mainline Protestants^23^. Additionally, several studies find that a higher degree of religiosity is correlated with lower environmental concern^23–25^. However, the role of religion in predicting pro-environmentalism varies across different religions. Some studies suggest that the relationship between religious beliefs and the rejection of human-caused climate change may not be directly causal^26^. Rather, the negative relationship between religious beliefs and pro-environment views and attitudes in the U.S. is likely due in part to the association between Christianity and political conservatism. It has been observed that invoking altruism effectively encourages the adoption of pro-environmental behaviors^27,28^.

Researchers have pointed out that the disparity in perspectives between experts and the broader population can be attributed to a lack of knowledge among the public^29^. Delli Carpini and Keeter^30^ contend that the extent of the public’s grasp on an issue significantly affects the caliber of discussions and the resulting policy reforms. Indeed, both Stoutenborough and Vedlitz^31^ and Guy et al.^32^ find that those with more objective knowledge generally express higher levels of concern regarding climate change. Finally, when it comes to conspiracy theories, recent studies find that higher beliefs or exposure to climate change conspiracy theories is associated with more pronounced anti-science attitudes, reduced concern for the environment, greater skepticism about climate change, and a diminished likelihood of participating in pro-environmental activities^33–36^.

While the literature tells us who should be more or less likely to engage in pro-environment behaviors, the novelty of our approach, which distinguishes between cynics and doubters, makes it harder to clearly predict if and how the subgroups behave differently. Based on this literature, we expect that being conservative, Republican, identifying as male, being older, worse-off financially, lower educated, religious, less knowledgeable on climate change, more likely to believe in conspiracy theories, and less altruistic should be correlated with being a doubter on sustainability behaviors. Since cynics believe that engaging in sustainability behaviors makes a difference for the environment, conservative political ideology, being Republican, lower environmental knowledge, and higher beliefs in conspiracy theories are plausibly less likely to be strong predictors of being a cynic.

To test our expectations we use individual-level survey data from the Caltech Climate Survey, conducted by YouGov using a nationally representative sample of 2096 U.S. registered voters in May 2022. Our findings from a set of ordinary least squares and multinomial logistic regressions reveal distinct characteristics associated with being cynics or doubters in relation to sustainability and climate change. In line with our expectations, doubters tend to have lower socioeconomic status, believe in conspiracy theories, exhibit lower altruism, possess limited knowledge about environmental concerns, identify as males, lack college education, and have voted for Trump while leaning towards conservative ideology. The influence of political variables is particularly pronounced. However, contrary to what we predicted, they are not more likely to be religious and they are more likely to be younger rather than older. On the other hand, cynics exhibit different traits. They tend to have higher socioeconomic status, possess greater environmental knowledge, are younger, hold religious beliefs, lean more liberal, and are less likely to have voted for Trump.

Our novel approach distinguishes between cynics and doubters, shedding light on the nuanced motivations and characteristics among these two groups of free-riders. By identifying these differences, our study aims to inform effective strategies for promoting climate-minded lifestyle changes.

## Data and methods

We use individual-level survey data from the Caltech Climate Survey. The survey was conducted by YouGov using a nationally representative sample of 2096 U.S. registered voters interviewed online between May 18–24, 2022. The data collection and analysis procedures were reviewed by the Institute Research Board at the California Institute of Technology, and were ruled exempt (IR22-1220). It is exempt under 45 C.F.R. §46.104(d)(2)(i),(ii) as it is research using survey procedures where the identity of the subjects cannot be readily ascertained. The interviewing and survey administration are conducted by YouGov, who provides us with a dataset with no identifying information for the subjects. In other words we receive data that is completely anonymized. The survey was carried out in accordance with relevant guidelines and regulations and informed consent was obtained from all participants.

Respondents were selected from YouGov’s opt-in panel to be representative of all U.S voters. For each study, YouGov draws a stratified random sample of its panelists to add to an invitation pool who are asked to take a survey. Those who respond to the invitation are routed to one of the open surveys. The algorithm that assigns responding panelists to surveys is intended to fill strata evenly over the field period using the target end date, elapsed time in field, sample size and definition, and priority.

For this study, the sample definition was self-reported active registered voters in the U.S., who were stratified on age (18–29, 30–44, 45–64, and 65+), race/ethnicity (white non-Hispanic, African American, Hispanic, and other), gender (male, female), education (high school or less, some college, college graduates, and post-graduate), and geographic region (Northeast, Midwest, South, West). The population targets for these strata come from model estimates by YouGov using the 2019 American Community Survey and November 2020 Current Population Survey, conducted by the U.S. Bureau of the Census, and TargetSmart Voter Files.

In order to develop weights to use for analysis of the survey data, YouGov used a raking procedure, based on gender, age, race, education, geographic region, and vote in the 2020 U.S. Presidential election^37^. The weights range from 0.3 to 5.05, with a mean of 1.0 and a standard deviation of 0.7. These weights are used in all analyses reported in this paper. The estimated margin of error for population proportions is approximately 2.6% (see “Supplementary Information (SI)” for more details on the sampling procedure). Table 1 provides weighted and unweighted descriptive statistics on key demographic variables (age, gender, education, region, and party identification).

This survey-based methodological approach is used extensively to study sustainable communities and public opinion regarding climate change^5,6,38^. In the next subsection we discuss in more detail the key survey questions that we use in our analysis. Additional details about survey data preprocessing are reported in the SI. The initial analyses use univariate and bivariate graphics to show the basic patterns of sustainability behavior among American registered voters. Then we use more sophisticated methods, in particular ordinary least squares and multinomial logistic regression, to estimate the multivariate results that we use to test our expectations for the relationships between the covariates in our dataset and the various types of sustainability-oriented behaviors we ask about in our surveys. We opted to employ ordinary least squares (OLS) models as our primary analytical approach instead of multinomial logistic regression models. Our decision was driven by the observation that the results obtained from both methods were not substantively different. Moreover, OLS provides the advantage of greater interpretability, allowing us to more easily communicate the relationships between variables, simplifying the presentation of our findings. We provide the detailed multinomial logistic regression results in the SI.

**Table 1:** Descriptive statistics, weighted and unweighted.

Variable	Unweighted descriptive statistics	Weighted descriptive statistics
	Overall	Overall
	N = 2096 (100.0%)	N = 2096 (100.0%)
Age		
65 or older	637 (30%)	558 (27%)
45 to 64	782 (37%)	732 (35%)
30 to 44	431 (21%)	483 (23%)
18 to 29	246 (12%)	322 (15%)
Gender		
Man	961 (46%)	954 (46%)
Woman	1,108 (53%)	1,109 (53%)
Other	27 (1.3%)	33 (1.6%)
Region		
Northeast	402 (19%)	373 (18%)
Midwest	480 (23%)	466 (22%)
South	781 (37%)	780 (37%)
West	433 (21%)	477 (23%)
Education		
HS or less	527 (25%)	639 (30%)
Some college	718 (34%)	614 (29%)
College grad	518 (25%)	532 (25%)
Postgrad	333 (16%)	311 (15%)
PartyID		
Democrat	758 (36%)	751 (36%)
Republican	577 (28%)	581 (28%)
Independent	636 (30%)	638 (30%)
Other	73 (3.5%)	74 (3.5%)
Not sure	52 (2.5%)	52 (2.5%)

### Sustainable behaviors

Our key dependent variables of interest are derived from two sets of questions about sustainable behaviors. The first set asks: in your everyday life, which of the following do you do to help protect the environment? Table 2 provides a list of the behaviors and frequencies of each answer.

**Table 2:** Sustainable behaviors: frequencies (%).

Sustainable behaviors	Don’t	Do
Compost kitchen waste	77.0	23.0
Eat less meat	73.0	27.0
Drive less or carpool	59.0	41.0
Reduce water usage	55.0	45.0
Reduce electricity use in the summer	51.5	48.5
Reduce food waste	46.5	53.5
Use fewer non-reusable plastics	46.1	53.9

We focus on these seven behaviors as they are relatively common ways that people may seek to live a more sustainable lifestyle. They are behaviors that have been included in previous polls of American opinions about climate change and sustainability^39^.

In the second set we ask individuals for each of the sustainable behaviors listed above: How much of a difference does it make when people do the following in their everyday lives to help protect the environment?

- A big difference
- A slight difference
- A very small difference
- No difference at all
- Not sure

### Sustainability believers, motivated, doubters, and cynics

Next, we try and categorize individuals based on whether they engage in these behaviors and whether they believe it makes or doesn’t make a big difference to engage in them (see Table 3). We call individuals who engage in these behaviors and think they make a big or slight difference “believers”; those who do not think they make a big or slight difference but engage in them “motivated”; and as described before, those who do not engage in these behaviors but believe they make a difference as “cynics”, while those that neither engage in them nor believe in them making a difference as “doubters”.

**Table 3:** Categories of individuals by whether they engage in sustainable behaviors and whether they think it makes a difference.

	I do it	I don’t do it
Big difference	Believers	Cynics
Slight difference	Believers	Cynics
Very small difference	Motivated	Doubters
No difference	Motivated	Doubters
Not sure	Motivated	Doubters

We focus here on the free-riders: the cynics and the doubters. Who are they? How do we change their behavior? To answer these questions we analyze the two groups separately and see what factors predict engaging in cynical and doubtful behaviors. We categorize respondents based on their engagement in sustainable behaviors and their beliefs about the impact of these behaviors on the environment. Specifically: Cynical behaviors refer to actions that respondents do not take but believe would positively impact the environment if they did. For example, if a respondent does not reduce electricity usage but believes that reducing electricity usage would make a difference for the environment, this non-action is classified as a cynical behavior for that individual. Doubtful behaviors, on the other hand, are actions that respondents neither undertake nor believe would make a significant environmental difference. This classification is tailored to each respondent’s unique set of responses. Thus, what constitutes a cynical behavior for one individual may be classified as a doubtful behavior for another, depending on their respective beliefs and actions. Our dependent variables are two variables ranging from 0 to 7 and they indicate, respectively, the number of cynical and doubtful behaviors the respondents engage in (in the SI we also analyze each behavior separately).

We want to see what variables are associated with cynical and doubtful behaviors. We look at different predictors: gender (binary 1 for woman and 0 otherwise), age group (Age 18–29, Age 30–44, Age 45 to 64, over 65), whether the respondent voted for Trump in the 2020 elections (binary 1 if they voted for Trump 0 otherwise), political ideology (1 very liberal to 7 very conservative), education (binary 1 if they have a 4-year college degree or more and 0 otherwise), subjective income (3 categories indicating whether the respondent is better off, the same, or worse off financially than a year ago), religious background (5 categories, including Protestant, Catholic, Jewish, other, and no religion), conspiracy theory score, altruism score, and environmental knowledge. To compute the last 3 measures (i.e., conspiracy theory score, altruism score, and environmental knowledge) we use Item Response Theory (IRT) models to reduce the dimensionality of the many survey responses regarding each of these three beliefs or opinions into a single scale (see the SI for more information on how these variables were calculated). We estimate our main models using ordinary least squares and we use multinomial logistic regressions in the SI to analyze each behavior separately. All models are weighted as discussed earlier.

### Ethical approval

The survey process was reviewed by Caltech’s Institutional Research Board (IRB 22-1220). Upon publication the code and data necessary to reproduce the results reported in this paper will be made available in a permanent and public data repository, subject to any limitations imposed by human subjects considerations.

## Results

### Descriptive analyses

**Figure 1:**
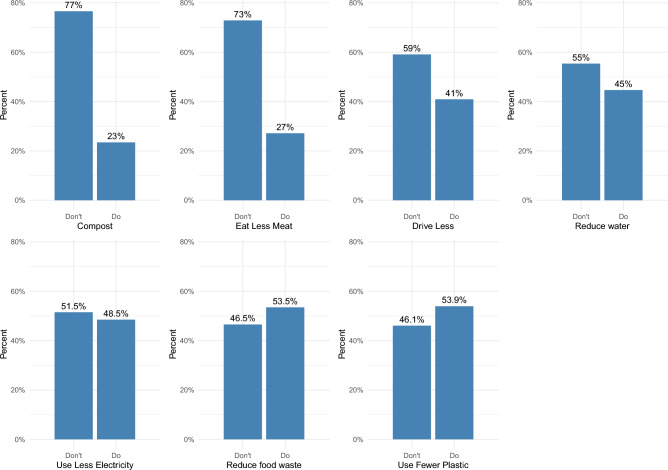
Percentage of people engaging in each sustainable behavior.

We can see the percentage of people engaging in each sustainable behavior in Fig. 1. There is variation in engagement with these behaviors, ranging from the 54% using fewer plastics that can’t be reused to only 23% composting kitchen waste.

**Figure 2:**
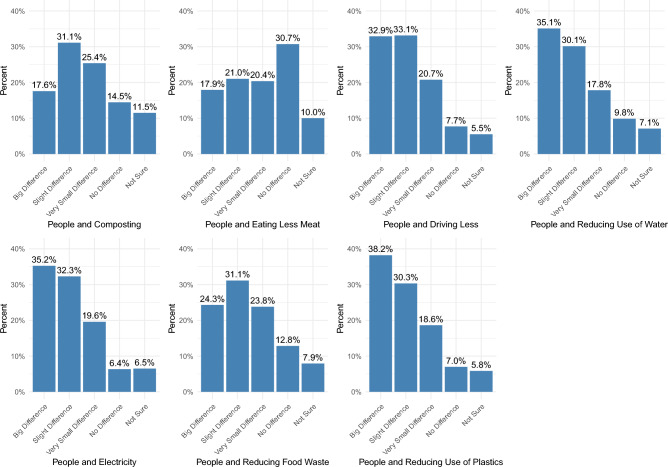
Percentage of people who think each sustainable behavior makes or does not make a difference.

Figure 2 also shows some variation in beliefs about whether these behaviors make a difference, as more individuals believe that behaviors like reducing plastic, water, and electricity use and driving less make more of a difference than eating less meat, reducing food waste, and composting.

**Figure 3:**
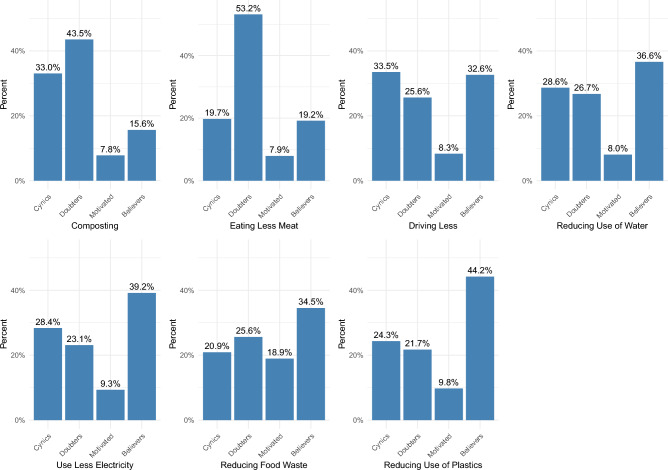
Percentage of people by category (cynics, doubters, motivated and believers) for each sustainable behavior.

Figure 3 shows that doubters (those that don’t believe these behaviors make a difference AND don’t engage in them) are a majority when it comes to eating less meat and composting. However, when we look at cynics and doubters together, these two groups constitute a majority on all behaviors except for two (reducing food waste and reducing the use of single-use plastics).

**Figure 4:**
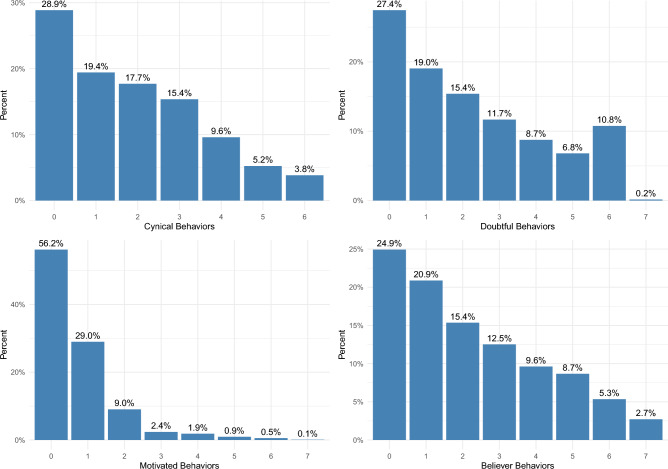
Percentage of people that engage in n (0 to 7) cynical, doubtful, motivated, or believer behaviors.

Are people consistently cynics, doubters, motivated, or believers? Not quite, as Fig. 4 illustrates. Specifically, it shows the number of cynical, doubtful, motivated, and believer behaviors the respondents engage in.

In the next section we use OLS and multinomial logistic regressions to investigate further what factors predict who will engage in either cynical or doubtful behaviors.

### Regression analyses

**Figure 5:**
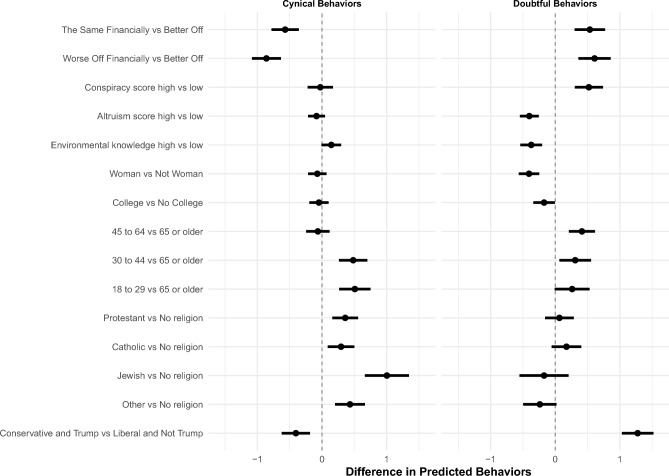
Differences in predicted engagement in cynical and doubtful behaviors across different quantities of interest.

**Figure 6:**
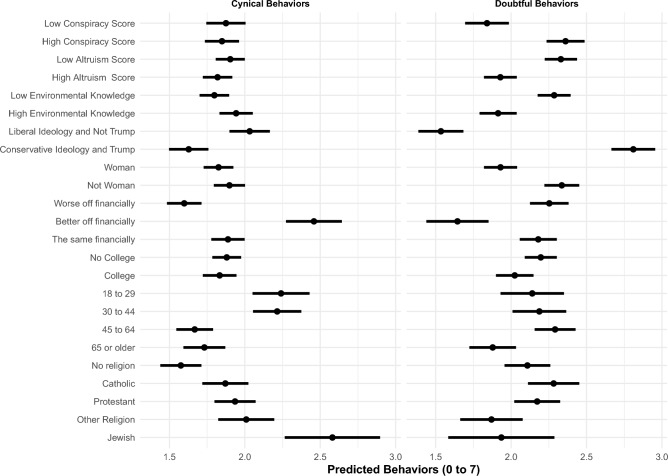
Predicted engagement in cynical and doubtful behaviors across different quantities of interest.

To help understand the substantive significance of our findings, after estimating two separate OLS regressions, one for cynical and one for doubtful behaviors, we present results in two ways^40^. As a reminder, our dependent variables are two variables ranging from 0 to 7 and they indicate, respectively, the number of cynical and doubtful behaviors the respondents engage in. First, in Fig. 5, we present differences in the predicted number of behaviors at different quantities of interest for each type of individual (cynics vs doubters). For continuous covariates, we show the difference in predicted number of behaviors associated with a change from the 10% to the 90% percentile across the sample. As an illustration, we might ask: If an individual were conservative and voted for Trump, but were otherwise unchanged in the value of her other covariates, how many more or less doubtful or cynical behaviors would she engage in compared to an individual who leans liberal and did not vote for Trump? In Fig. 6 we show the predicted number of behaviors at different quantities of interest from the OLS regression. To illustrate this, it would be akin to asking: If an individual were conservative and voted for Trump, but were otherwise unchanged in the value of her other covariates, how many doubtful or cynical behaviors would she engage in on average?

What emerges from a first glance at the comparisons in the predicted number of behaviors at different quantities of interest is that doubters and cynics are very different (see Figs. 5 and 6). People who are less well-off financially, who believe in conspiracy theories more, who are less altruistic, who have lower knowledge about environmental issues, who identify as males, who did not go to college, who are relatively younger, and who voted for Trump and identify as more conservative engage in more doubtful behaviors, meaning that they are less likely to engage in sustainable behaviors and less likely to believe these behaviors make a difference. Contrary to what we predicted, they are not more likely to be religious and they are more likely to be younger rather than older. The political variables produce the strongest association by far: being conservative and voting for Trump is associated with 1.5 more doubtful behaviors relative to someone who’s liberal and did not vote for Trump. In other words, this means that those who are conservative and those voting for Trump are those who are engaging in fewer sustainable behaviors and who do not believe they make a difference.

Cynics appear overall to be slightly different on most characteristics. People who are more well-off financially, who have higher environmental knowledge, who are younger, who are religious, and who are more liberal and did not vote for Trump are more likely to engage in more cynical behaviors, meaning they are less likely to engage in sustainability behaviors, while believing these behaviors would make a difference.

In the SI we show results from multinomial logistic regressions for each type of behavior (we predict the probability of being believers, motivated, doubters, or cynics for each behavior). When we look at the multinomial logistic results for each individual behavior and focus on cynics and doubters we see fairly similar results compared to the OLS on the sum of behaviors, with some variation by behavior especially among cynics. On aggregate the most consistent finding is the political one for doubters: doubters are much more likely to be conservative and have voted for Trump; when it comes to eating less meat a conservative who also voted for Trump is almost 40% more likely to be a doubter than a liberal who did not vote for Trump. Furthermore, across behaviors, doubters are worse-off financially, have higher conspiracy scores, lower altruism, lower environmental knowledge, and are more likely to be male and younger. There is a bit more variation for cynics across behaviors: the one consistent finding is that they tend overall to be better-off financially and for most behaviors they are slightly younger, more religious, more liberal and did not vote for Trump.

### Discussion

This paper studies the factors that predict engagement in sustainable behaviors among American voters, focusing on two distinct subgroups of free-riders: cynics and doubters. By differentiating between those who do not engage in sustainability behaviors but recognize their importance (cynics) and those who neither engage in nor acknowledge the significance of these actions (doubters), we uncover valuable insights into their motivations and socio-demographic characteristics.

Our findings highlight that cynics, who believe in the importance of sustainability behaviors but don’t engage in them, tend to have higher socioeconomic status, possess greater environmental knowledge, are more religious, lean more liberal, and are less likely to have voted for Trump. On the other hand, doubters, who neither recognize the importance of sustainability behaviors nor engage in them, exhibit lower socioeconomic status, believe in conspiracy theories, possess limited knowledge about environmental concerns, identify as males, lack college education, and voted for Trump while leaning towards conservative ideology (as shown in Figs. 5 and 6).

These distinct demographic profiles of cynics and doubters underscore the need for targeted interventions to encourage behavior change. Strategies aimed at cynics could focus on leveraging their existing knowledge, and their religious and liberal leanings, while interventions for doubters could address their barriers related to socioeconomic status, conspiracy beliefs, and limited environmental knowledge.

## Conclusion

In our study involving 2096 registered voters in the United States, we delved into the sustainability behaviors of two distinct groups of environmental free riders. The first group, cynics, acknowledges the importance of environmental issues yet refrains from engaging in sustainable practices. The second group, doubters, neither acknowledges the importance of these issues nor participates in sustainable actions. We found that doubters generally tend to be younger males, often with lower income and educational backgrounds. This group exhibits a stronger inclination towards conspiracy theories, lower levels of altruism, low environmental knowledge, and a tendency to support Trump and conservative ideologies. Conversely, cynics are typically younger, more religious, enjoy a higher socioeconomic status, are well-informed about environmental matters, lean towards liberal ideologies, and are less likely to be Trump supporters.

Overall, a critical implication of our research is the advancement of our understanding of free-riders and their role in climate change mitigation, which can help policymakers, educators, and environmental activists design tailored interventions to promote a climate-sensitive lifestyle. In this respect, we can combine Olson’s more top-down approach, highlighting selective incentives, with Ostrom’s bottom-up principles, underscoring community-driven initiatives, to develop effective strategies to address the issue of free-riders in environmental behavior^8,41^.

For doubters, who are more likely to be financially constrained, incentivizing sustainability through positive incentives, such as financial rewards, may be particularly effective. Indeed, monetary incentives have proven particularly effective at promoting pro-environmental behaviors across different contexts^42–46^. Furthermore, educational and awareness initiatives about the individual, as well as the collective net benefits of engaging in sustainability behaviors, may successfully address both doubters’ efficacy concerns and knowledge gaps. Empirical evidence suggests that the effectiveness of education and awareness methods on sustainable behaviors varies widely, from minimal impact with basic information dissemination to significantly better outcomes with tailored information and public pledges. Top-down directives are notably ineffective, highlighting the need for more personalized and engaging approaches to induce behavior change^42^. Increasingly, the literature also shows that conspiracy beliefs can be addressed to a certain extent through psychological inoculation, which can also be effective in motivating doubters toward sustainable behavior.

Meanwhile, cynics, who are aware of but inactive in sustainable practices, may be motivated through more community-driven and or social influence initiatives. Close social groups such as friends and members of one’s community can exert social influence on one’s behavior^42,47^. This approach, based on the assumption that information provision will be more effective when conveyed by somebody from the same social network, has been effective at encouraging resource conservation^42,47^. Given cynics’ higher likelihood of being religious and liberal, engaging them in local environmental management and decision-making, particularly through channels aligned with their religious or political affiliations, could effectively translate their passive beliefs into active participation. These different approaches acknowledge the diverse motivations and barriers within these groups, aiming to foster a more inclusive and effective environmental stewardship.

This area of research needs future study. We need to push further to understand more about the motivations of the free riders so as to better determine how to persuade them to engage more in a sustainable lifestyle. Future research should explore a wider array of sustainable behaviors, and scholars should start studying what types of arguments might *cause* changes in behavior, perhaps following the example of recent “deep persuasion” research^48^.

Observational studies like ours are important to establish the types of sustainable behaviors and the target populations and can be paired with detailed causal field experiments that will have strong internal validity. One novel approach would be to manipulate people’s willingness to engage in sustainability behaviors by implementing a prisoner’s dilemma game with real payoffs. This study design, by immersing respondents in this interactive scenario, would allow us to test whether experiencing the consequences of free-riding behavior more practically could enhance respondents’ understanding of the collective action problem and prompt behavioral changes. This type of experimental manipulation could shed further light on the decision-making processes of cynics and doubters, offering valuable insights into the efficacy of different strategies for promoting sustainable actions.

### Supplementary Information

Supplementary Information.

## Data Availability

Upon publication the code and data necessary to reproduce the results reported in this paper will be made available in a permanent and public data repository, subject to any limitations imposed by human subjects considerations. Replication materials can be accessed here: https://drive.google.com/drive/folders/1oAOnYajSWRkA_WU5fQAOU8SXmD-6z9be.
