# Supplementary material for: Identifying American climate change free riders and motivating sustainable behavior
Source: Sci Rep. 2024 Mar 19;14:6575. doi: 10.1038/s41598-024-57042-w (PMC10951196; doi:10.1038/s41598-024-57042-w)
Supplement: Supplementary file 1 — Supplementary Information. [file 41598_2024_57042_MOESM1_ESM.pdf]

Supplementary Information: Identifying American Climate Change Free Riders and Motivating Sustainable Behavior

1. Data and Methods

1.1. Survey and Sampling

This supplementary material contains additional information on our data. Our questions come from the Caltech Climate Survey, which was conducted by YouGov using an online nationally representative sample of 2,096 US registered voters between May 18-24, 2022.

This sample was made representative of all U.S. residents by applying weights based on gender, age, race, education (derived from the American Community Survey by the U.S. Bureau of the Census), and 2020 Presidential vote.

The weights range from 0.3 to 5.05, with an average of 1.0 and a standard deviation of 0.7 (see figure S1). To account for sampling variability, the margin of error (a 95% confidence interval) for a sample percentage p calculated from the entire sample is approximately 2.6%. This margin of error is computed using the formula:

$$\widehat{p} \pm 100 \times \sqrt{\frac{1 + CV^2}{n}} \tag{1}$$

where CV represents the coefficient of variation of the sample weights, and n is the sample size used to compute the proportion. This measurement helps assess sampling error, reflecting how much the sample estimate is expected to differ from its true population value in 95% of all samples. However, it does not account for non-sampling errors, such as potential biases in panel participation or survey response.

1.2. Independent variables

The main manuscript contains information on how we derived our key dependent variables. This section contains information on the following independent variables that were used in our analyses: gender, age, whether the respondent voted for Trump in the 2020 elections, political ideology, education, subjective income, religious background, conspiracy theory score, altruism score, and environmental knowledge score.

We created a binary variable *woman*, taking value 1 if the respondent identifies as a woman and 0 otherwise based on the following question:

Gender: Which gender identity do you most identify with? (Allows one selection).

- Woman

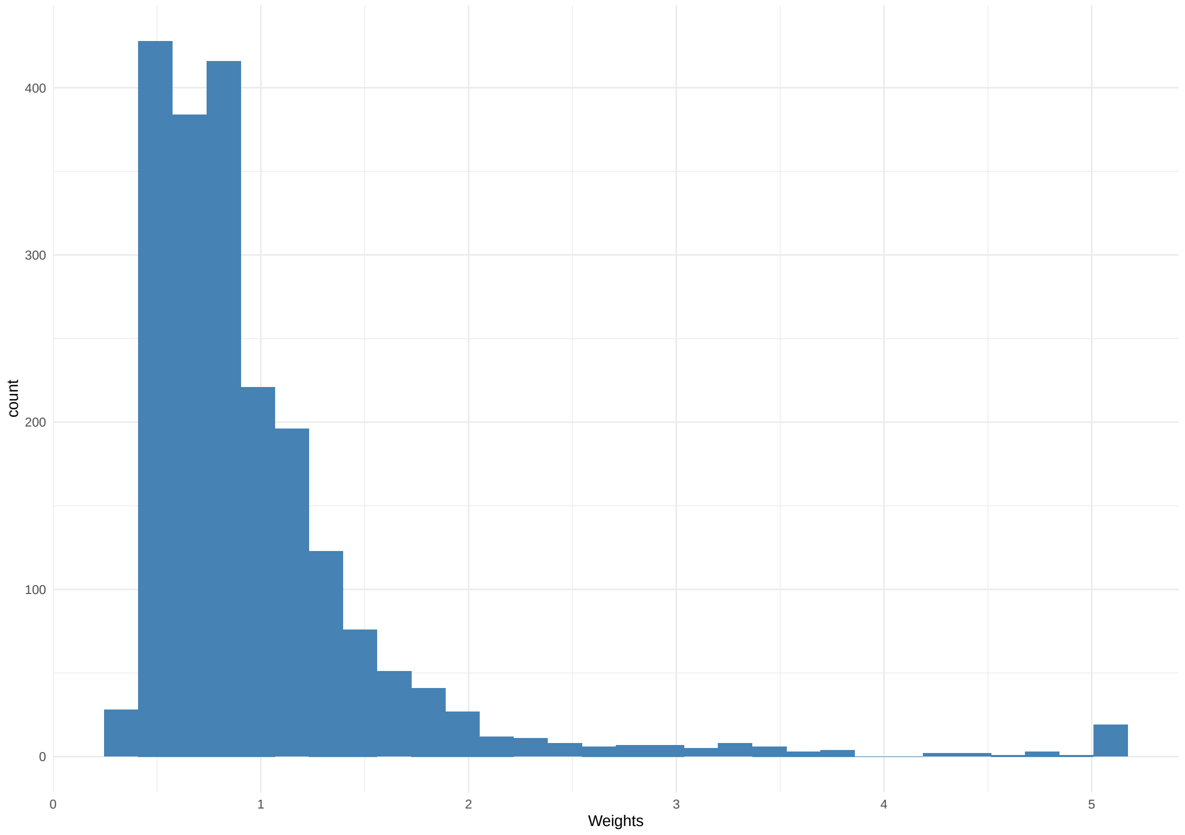

Figure S1. Distribution of weights

- Man
- Non-Binary/Fluid
- Prefer not to say

The variable *age group* (Age 18-29, Age 30-44, Age 45 to 64, over 65) was calculated from birth year:

Birth Year: In what year were you born?

Trump is a binary variable that takes value 1 if the respondent voted for Trump and 0 otherwise based on the following question:

Trump: In the 2020 presidential election, did you vote for (Allows one selection).

- Joe Biden
- Donald Trump
- Someone else
- Didn't vote

After excluding don't knows, *political ideology* takes values 1 to 7 based on the following question:

Political Ideology: In politics today, do you generally think of yourself as... (Allows one selection)

- Extremely liberal
- Liberal
- Slightly liberal
- Moderate
- Slightly conservative
- Conservative
- Extremely conservative

Education takes value 1 if the respondent has a 4-year college degree or more and 0 otherwise and is based on the following question:

Education: What is the highest level of education you have completed? (Allows one selection)

- Did not graduate from high school
- High school graduate
- Some college, but no degree (yet)
- 2-year college degree
- 4-year college degree
- Postgraduate degree (MA, MBA, MD, JD, PhD, etc.)

The variable *subjective income* has 3 categories based on the following question:

Subjective Income: We are interested in how people are getting along financially these days. Would you say that you and your family living here are better off or worse off financially than you were a year ago? (Allows one selection)

- Better off
- The same
- Worse off

The variable *religion* has 5 categories (Protestant, Catholic, Jewish, Other, No religion) and is based on the following question :

Religion: What is your religious preference? Is it Protestant, Catholic, Jewish, Muslim, some other religion, or no religion? (Allows one selection)

- Protestant
- Catholic
- Jewish
- Muslim
- Some other religion
- No religion

In our survey, we included a wide range of questions that we use to form variables for our models to measure an individual's propensity to believe in conspiracy theories, to measure the extent to which they are altruistic, and to measure their domain-specific environmental knowledge. So as to save degrees of freedom in our models, and to make our model specifications more parsimonious, we use Item Response Theory (IRT) models to reduce the dimensionality of the many survey responses regarding each of these three beliefs or opinions into single scales. IRT models are commonly used in the social sciences for modeling how items (here answers to survey questions) are correlated with an underlying latent variable.†

We employed a Graded Response Model, a type of polytomous Item Response Theory (IRT) model, to assess individuals' conspiracy theory beliefs. Our analysis involved a set of 26 questions, with responses measured on a graded scale. By utilizing this model, we generated a robust conspiracy theory belief score that captures the nuanced levels of agreement or disagreement expressed across the questionnaire. We used the grm() function in the ltm package in R to create the score. These are the 26 questions we used to create the *conspiracy theory score*. All responses go from 1 strongly disagree to 7 strongly agree:

- Conspiracy theory beliefs 1: The rapid spread of certain viruses and/or diseases is the result of the deliberate, concealed efforts of some organization.
- Conspiracy theory beliefs 2: Groups of scientists deliberately attempt to create panic about future risks because it is in their interest to do so.
- Conspiracy theory beliefs 3: Many well- known celebrities, politicians, and wealthy people are members of a secret society, which has control over our lives.
- Conspiracy theory beliefs 4: The United Nations is part of the new world order, planning to undermine American sovereignty and aiming to introduce a global currency to end US dollar.
- Conspiracy theory beliefs 5: Governments use COVID-19 testing to collect genetic information about their citizens.

†For an excellent introduction to ITR, see Simon Jackman, "Measurement", in *The Oxford Handbook of Political Methodology*, edited by Janet M. Box-Steffensmeier, Henry E. Brady and David Collier, Oxford University Press, 2008, pages 119-51.

- Conspiracy theory beliefs 6: Small groups of people are in possession of secret knowledge which would change our understanding of the world and are deliberately keeping it hidden.
- Conspiracy theory beliefs 7: Agenda 21, the United Nations sustainable development plan, encompasses the expropriation of citizens and taking away personal freedom as part of their sustainability plan.
- Conspiracy theory beliefs 8: Experiments involving advanced technologies are carried out on the general public without their knowledge or consent.
- Conspiracy theory beliefs 9: Cures for certain deadly common diseases exist, but are deliberately withheld.
- Conspiracy theory beliefs 10: The government is manipulating official laboratory tests and techniques, so that it looks like a lot of people have fallen victim to COVID-19.
- Conspiracy theory beliefs 11: The power held by heads of state is second to that of small unknown groups who really control world politics.
- Conspiracy theory beliefs 12: Unbeknownst to the general public global elites take part in torturing children to harvest a chemical compound that promises youthfulness and health forever.
- Conspiracy theory beliefs 13: The condensation trails from aircraft actually contain toxic chemicals, as part of secretive government actions.
- Conspiracy theory beliefs 14: Some movies give hints about how powerful groups control the world and act to harm children.
- Conspiracy theory beliefs 15: Drugs (such as vaccines) are deliberately supplied to (certain) communities in order to marginalize or destroy them.
- Conspiracy theory beliefs 16: HIV was invented and purposefully distributed by the CIA.
- Conspiracy theory beliefs 17: Some of the people thought to be responsible for acts of terrorism were actually set up by those in charge.
- Conspiracy theory beliefs 18: A certain event, such as a global pandemic, natural disaster or terrorist attack will allow the government and people in charge to impose martial law, leading to the seizure of arms, travel bans and detainment of citizens.
- Conspiracy theory beliefs 19: The government has staged important societal events in order to manipulate voters.
- Conspiracy theory beliefs 20: Government agencies have been secretly involved in the assassination of their own citizens.
- Conspiracy theory beliefs 21: Some viruses and/or diseases which many people are infected with were created in laboratories as bio-weapons.
- Conspiracy theory beliefs 22: Malaysian Airlines flight MH17 was purposefully shot down to conceal sensitive information that could have been spread by passengers on board.

- Conspiracy theory beliefs 23: The government has ordered the manipulation of official laboratory PCR tests so that less people will test positive for COVID-19.
- Conspiracy theory beliefs 24: In the 2020 election, non-citizen voting and voting of dead people in some states changed the outcome.
- Conspiracy theory beliefs 25: Novel messenger RNA vaccines cannot alter people's genome and cause death or disease
- Conspiracy theory beliefs 26: Certain celebrities and/or public figures actually faked their own death in order to escape the spotlight.

We created the *altruism score* in a similar way as the conspiracy theory belief score using an IRT model based on 10 altruism questions in the survey. These are the 10 questions we used to create the score:

- Altruism 1: Please indicate the frequency with which you have carried out the following acts [Never, Once, More than once, Often, Very Often]
- Altruism 2: I have given money to a charity [Never, Once, More than once, Often, Very Often]
- Altruism 3: I have helped an acquaintance to move households [Never, Once, More than once, Often, Very Often]
- Altruism 4: I have before being asked, voluntarily looked after a neighbor's pets or children without being paid for it [Never, Once, More than once, Often, Very Often]
- Altruism 5: I have donated goods or clothes to a charity [Never, Once, More than once, Often, Very Often]
- Altruism 6: I have offered my seat on a bus or train to a stranger who was standing [Never, Once, More than once, Often, Very Often]
- Altruism 7:I have given money to stranger who needed it or asked me for it [Never, Once, More than once, Often, Very Often]
- Altruism 8: I have donated blood [Never, Once, More than once, Often, Very Often]
- Altruism 9: I have helped carry a stranger's belongings (books, bags, etc.) [Never, Once, More than once, Often, Very Often]
- Altruism 10: I have let a neighbor whom I didn't know too well borrow some item of value to me (tools, dishes, etc.) [Never, Once, More than once, Often, Very Often]

Finally, we created the *environmental knowledge score* in a similar way as the conspiracy theory belief score and the altruism score using an IRT model based on 5 environmental knowledge questions in the survey. We use the ltm() function in R to create the environmental knowledge score, since this function fits a latent trait model for binary data. These are the 5 questions we used to create the score:
Environmental knowledge 1: The global average temperature increased between 1901 and 2016 by: (Allows one selection)

- 1.1°F
- 1.8°F [correct]
- 2.5°F
- 5°F

Environmental knowledge 2: How many weather- and climate-related billion-dollar disasters did the U.S. experience in 2020?(Allows one selection)

- 5
- 12
- 20 [correct]
- 35

Environmental knowledge 3: What was the costliest hurricane in U.S. history? (Allows one selection)

- Katrina [correct]
- Harvey
- Sandy
- Andrew

Environmental knowledge 4: The sea level along the U.S. coastline is predicted to rise in the next 30 years by: (Allows one selection)

- 2-3 inches
- 5-6 inches
- 10-12 inches [correct]
- 24-26 inches

Environmental knowledge 5: Is the United States a party to the Paris Agreement, an international treaty seeing to limit global warming? (Allows one selection)

- Yes [correct]
- No

1.2.1. Who are Trump voters: Here we show what individual characteristics are correlated with voting for Trump, which is one of our explanatory variables. Figure S2 shows that Trump voters are older, more likely to be male, less likely to have a college degree, more likely to be worse-off financially, more likely to be white, religious, conservative, and Republican.

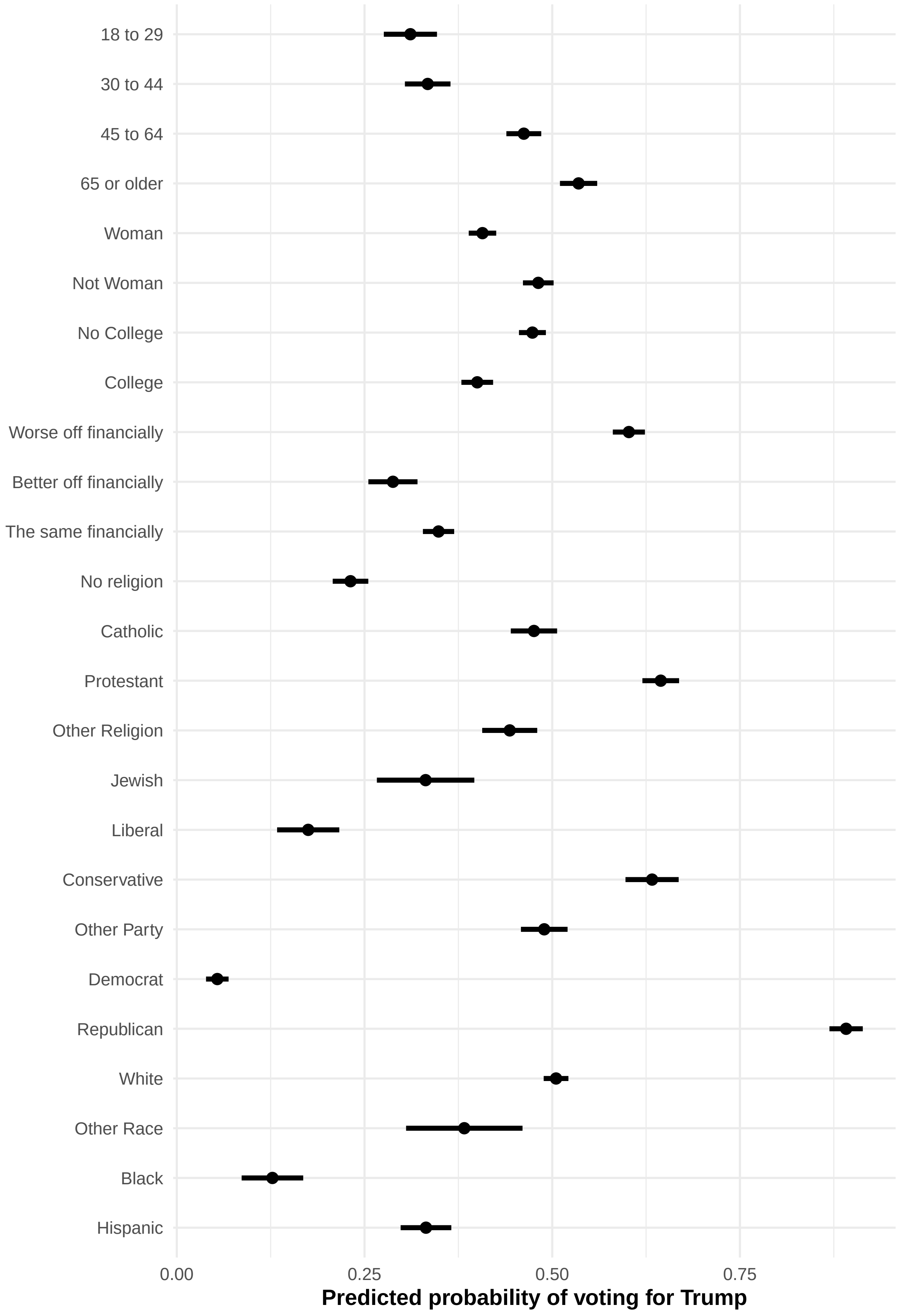

Figure S2. Predicted probability of voting for Trump by various demographic characteristics

2. Regressions

In this study, all statistical models were fitted using the R programming language. The Ordinary Least Squares (OLS) models and Multinomial Logit Regressions (MLR) were run using the lm() and multinom() functions in R, respectively. To ensure the representativeness of our sample, we applied the weights discussed above to all of our regression models. In addition, we used listwise deletion to handle missing data in all analyses. This method excluded cases with missing values in any of the variables used in the respective models. To plot all of our figures, we utilized the marginaleffects() package in R to produce post-estimation predicted values and comparisons across quantities of interest.

Table S1. Weighted OLS regressions on number of cynical and doubtful behaviors. Standard errors are given in parentheses.

	Dependent variable:	
	Doubtful Behaviors	Cynical Behaviors
	(1)	(2)
Conspiracy Theory Score	0.230***	−0.012
	(0.049)	(0.044)
Environ. Knowledge Score	−0.187***	0.072
	(0.043)	(0.039)
Altruism Score	−0.200***	−0.042
	(0.037)	(0.033)
Voted for Trump	0.913***	−0.429***
	(0.114)	(0.102)
Woman	−0.406***	−0.072
	(0.081)	(0.073)
The same financially (ref. Better off)	0.536***	−0.570***
	(0.120)	(0.108)
Worse off financially	0.609***	−0.860***
	(0.127)	(0.114)
College Education or More	−0.173*	−0.047
	(0.085)	(0.076)
Age 45 to 64 (ref. over 65)	0.413***	−0.064
	(0.103)	(0.093)
Age 30 to 44	0.309*	0.483***
	(0.125)	(0.112)
Age 18 to 29	0.262	0.508***
	(0.138)	(0.124)
Political ideology	0.091**	0.006
	(0.031)	(0.028)
Protestant (ref. No religion)	0.065	0.359***
	(0.114)	(0.102)
Catholic	0.174	0.295**
	(0.117)	(0.105)
Jewish	−0.173	1.005***
	(0.194)	(0.174)
Other religion	−0.238	0.434***
	(0.132)	(0.118)
Constant	0.793***	2.304***
	(0.198)	(0.178)
Observations	2,005	2,005
R^2	0.219	0.129
Note:	* p<0.05; ** p<0.01; *** p<0.001	

Table S2. Weighted multinomial logistic regressions by type on use of electricity (ref. Believers): Odds ratios, confidence intervals and p-values.

	**Cynics**			**Doubters**			**Motivated**		
**Characteristic**	**OR**	**95% CI**	**p-value**	**OR**	**95% CI**	**p-value**	**OR**	**95% CI**	**p-value**
Conspiracy Theory Score	1.13	0.99, 1.30	0.079	1.55	1.33, 1.82	¡0.001	1.29	1.04, 1.59	0.020
Environ. Knowledge Score	0.93	0.83, 1.05	0.2	0.72	0.62, 0.83	¡0.001	0.85	0.71, 1.03	0.10
Altruism Score	0.83	0.75, 0.92	¡0.001	0.78	0.70, 0.88	¡0.001	0.81	0.69, 0.95	0.010
Voted For									
Not Trump	—	—		—	—		—	—	
Trump	1.69	1.22, 2.34	0.002	2.62	1.85, 3.73	¡0.001	2.44	1.51, 3.93	¡0.001
Gender									
Not Woman	—	—		—	—		—	—	
Woman	0.60	0.48, 0.76	¡0.001	0.59	0.46, 0.76	¡0.001	0.82	0.59, 1.16	0.3
Subjective Income									
Better off	—	—		—	—		—	—	
The same	0.80	0.58, 1.10	0.2	1.98	1.32, 2.98	0.001	1.82	1.01, 3.30	0.048
Worse off	0.59	0.42, 0.84	0.003	1.52	0.99, 2.33	0.054	2.04	1.11, 3.73	0.021
Education									
No College	—	—		—	—		—	—	
College	1.08	0.85, 1.37	0.5	0.93	0.72, 1.21	0.6	1.00	0.70, 1.43	¿0.9
Age									
65 or older	—	—		—	—		—	—	
45 to 64	1.00	0.75, 1.34	¿0.9	1.84	1.33, 2.55	¡0.001	1.83	1.18, 2.85	0.007
30 to 44	1.81	1.28, 2.57	¡0.001	2.86	1.92, 4.25	¡0.001	2.60	1.51, 4.46	¡0.001
18 to 29	2.01	1.36, 2.95	¡0.001	3.41	2.20, 5.29	¡0.001	2.93	1.61, 5.32	¡0.001
Political ID	0.91	0.83, 1.0	0.038	0.96	0.87, 1.07	0.5	0.93	0.82, 1.07	0.3
Religion									
No religion	—	—		—	—		—	—	
Protestant	1.05	0.76, 1.44	0.8	0.93	0.65, 1.32	0.7	0.92	0.57, 1.47	0.7
Catholic	1.01	0.73, 1.42	¿0.9	1.21	0.85, 1.73	0.3	0.89	0.54, 1.46	0.6
Jewish	4.62	2.47, 8.63	¡0.001	2.76	1.32, 5.76	0.007	3.05	1.25, 7.44	0.014
Other	0.95	0.66, 1.36	0.8	0.60	0.39, 0.91	0.017	0.67	0.39, 1.17	0.2

[1] OR = Odds Ratio, CI = Confidence Interval

Table S3. Weighted multinomial logistic regressions by type on meat consumption (ref. Believers): Odds ratios, confidence intervals and p-values.

	Cynics			Doubters			Motivated		
**Characteristic**	**OR**	**95% CI**	**p-value**	**OR**	**95% CI**	**p-value**	**OR**	**95% CI**	**p-value**
Conspiracy Theory Score	1.11	0.92, 1.33	0.3	1.32	1.12, 1.56	¡0.001	1.35	1.07, 1.71	0.013
Environ. Knowledge Score	1.14	0.98, 1.32	0.091	1.02	0.89, 1.17	0.8	0.91	0.74, 1.12	0.4
Altruism Score	0.93	0.81, 1.07	0.3	0.80	0.70, 0.90	¡0.001	1.07	0.90, 1.27	0.5
Voted For									
Not Trump	—	—		—	—		—	—	
Trump	1.31	0.83, 2.07	0.2	3.76	2.52, 5.62	¡0.001	1.90	1.08, 3.33	0.025
Gender									
Not Woman	—	—		—	—		—	—	
Woman	0.73	0.54, 0.99	0.040	0.64	0.49, 0.84	0.001	1.09	0.73, 1.62	0.7
Subjective Income									
Better off	—	—		—	—		—	—	
The same	0.95	0.65, 1.39	0.8	1.67	1.14, 2.46	0.009	1.75	0.99, 3.08	0.052
Worse off	0.76	0.49, 1.16	0.2	2.10	1.40, 3.16	¡0.001	1.67	0.92, 3.06	0.093
Education									
No College	—	—		—	—		—	—	
College	0.68	0.50, 0.93	0.014	0.62	0.47, 0.81	¡0.001	0.71	0.48, 1.06	0.10
Age									
65 or older	—	—		—	—		—	—	
45 to 64	1.18	0.78, 1.78	0.4	1.52	1.08, 2.13	0.016	0.95	0.57, 1.58	0.8
30 to 44	2.54	1.59, 4.05	¡0.001	1.29	0.85, 1.97	0.2	1.72	0.96, 3.07	0.068
18 to 29	2.22	1.36, 3.60	0.001	1.00	0.64, 1.58	¿0.9	1.43	0.76, 2.68	0.3
Political ID	1.14	1.02, 1.28	0.018	1.29	1.16, 1.43	¡0.001	1.12	0.97, 1.30	0.13
Religion									
No religion	—	—		—	—		—	—	
Protestant	1.79	1.17, 2.73	0.007	1.39	0.95, 2.03	0.087	1.44	0.85, 2.46	0.2
Catholic	1.73	1.13, 2.65	0.012	1.36	0.93, 2.00	0.12	1.15	0.66, 2.00	0.6
Jewish	3.30	1.72, 6.34	¡0.001	1.24	0.63, 2.44	0.5	1.13	0.41, 3.11	0.8
Other	0.84	0.52, 1.34	0.5	0.71	0.47, 1.07	0.10	0.54	0.29, 1.03	0.060

[1] OR = Odds Ratio, CI = Confidence Interval

Table S4. Weighted multinomial logistic regressions by type on driving behavior (ref. Believers): Odds ratios, confidence intervals and p-values.

	Cynics			Doubters			Motivated		
**Characteristic**	**OR**	**95% CI**	**p-value**	**OR**	**95% CI**	**p-value**	**OR**	**95% CI**	**p-value**
Conspiracy Theory Score	1.04	0.91, 1.20	0.6	1.43	1.21, 1.67	¡0.001	1.35	1.08, 1.70	0.009
Environ. Knowledge Score	0.98	0.87, 1.10	0.8	0.89	0.77, 1.03	0.12	1.00	0.83, 1.22	¿0.9
Altruism Score	0.83	0.74, 0.92	¡0.001	0.71	0.62, 0.80	¡0.001	0.78	0.66, 0.92	0.004
Voted For									
Not Trump	—	—		—	—		—	—	
Trump	1.21	0.87, 1.67	0.3	3.02	2.10, 4.34	¡0.001	1.52	0.92, 2.52	0.10
Gender									
Not Woman	—	—		—	—		—	—	
Woman	1.04	0.83, 1.31	0.7	0.77	0.60, 1.00	0.051	0.72	0.50, 1.03	0.076
Subjective Income									
Better off	—	—		—	—		—	—	
The same	1.00	0.72, 1.39	¿0.9	1.94	1.28, 2.93	0.002	1.17	0.67, 2.03	0.6
Worse off	0.75	0.53, 1.05	0.10	1.42	0.92, 2.19	0.11	1.17	0.66, 2.06	0.6
Education									
No College	—	—		—	—		—	—	
College	1.18	0.93, 1.49	0.2	1.07	0.81, 1.40	0.6	1.12	0.77, 1.63	0.6
Age									
65 or older	—	—		—	—		—	—	
45 to 64	1.36	1.03, 1.80	0.032	2.13	1.53, 2.97	¡0.001	1.53	0.96, 2.43	0.071
30 to 44	2.64	1.84, 3.78	¡0.001	4.65	3.08, 7.02	¡0.001	1.86	1.02, 3.41	0.044
18 to 29	2.28	1.55, 3.35	¡0.001	2.74	1.73, 4.35	¡0.001	3.02	1.68, 5.41	¡0.001
Political ID	1.15	1.05, 1.25	0.002	1.15	1.04, 1.27	0.008	1.26	1.09, 1.45	0.002
Religion									
No religion	—	—		—	—		—	—	
Protestant	1.11	0.81, 1.51	0.5	1.47	1.01, 2.13	0.043	0.52	0.31, 0.87	0.012
Catholic	1.51	1.08, 2.09	0.015	1.92	1.30, 2.83	0.001	1.08	0.66, 1.76	0.8
Jewish	0.69	0.39, 1.21	0.2	1.62	0.88, 2.99	0.12	0.80	0.35, 1.80	0.6
Other	1.01	0.70, 1.45	¿0.9	1.14	0.75, 1.74	0.5	0.44	0.23, 0.82	0.010

[1] OR = Odds Ratio, CI = Confidence Interval

Table S5. Weighted multinomial logistic regressions by type on food waste (ref. Believers): Odds ratios, confidence intervals and p-values.

	Cynics			Doubters			Motivated		
**Characteristic**	**OR**	**95% CI**	**p-value**	**OR**	**95% CI**	**p-value**	**OR**	**95% CI**	**p-value**
Conspiracy Theory Score	1.17	1.01, 1.37	0.043	1.28	1.10, 1.49	0.001	1.16	0.99, 1.37	0.070
Environ. Knowledge Score	0.91	0.80, 1.04	0.2	0.71	0.62, 0.82	¡0.001	0.91	0.79, 1.05	0.2
Altruism Score	0.82	0.73, 0.92	¡0.001	0.82	0.73, 0.92	¡0.001	0.83	0.74, 0.94	0.004
Voted For									
Not Trump	—	—		—	—		—	—	
Trump	1.08	0.76, 1.54	0.7	1.84	1.30, 2.59	¡0.001	2.81	1.93, 4.10	¡0.001
Gender									
Not Woman	—	—		—	—		—	—	
Woman	0.69	0.54, 0.90	0.005	0.58	0.45, 0.74	¡0.001	0.69	0.53, 0.90	0.006
Subjective Income									
Better off	—	—		—	—		—	—	
The same	0.63	0.45, 0.88	0.008	1.38	0.93, 2.05	0.11	1.48	0.96, 2.29	0.074
Worse off	0.45	0.31, 0.66	¡0.001	1.36	0.90, 2.05	0.14	1.15	0.73, 1.80	0.6
Education									
No College	—	—		—	—		—	—	
College	0.94	0.72, 1.23	0.6	0.88	0.68, 1.14	0.3	0.78	0.59, 1.03	0.084
Age									
65 or older	—	—		—	—		—	—	
45 to 64	1.50	1.06, 2.13	0.024	1.36	1.00, 1.85	0.049	1.57	1.13, 2.18	0.007
30 to 44	1.81	1.22, 2.70	0.003	1.29	0.89, 1.88	0.2	1.08	0.71, 1.64	0.7
18 to 29	2.26	1.47, 3.46	¡0.001	1.28	0.83, 1.97	0.3	1.50	0.96, 2.36	0.078
Political ID	1.01	0.91, 1.11	¿0.9	1.08	0.98, 1.19	0.13	1.00	0.90, 1.11	¿0.9
Religion									
No religion	—	—		—	—		—	—	
Protestant	1.92	1.33, 2.79	¡0.001	1.41	1.00, 1.99	0.050	1.00	0.69, 1.43	¿0.9
Catholic	1.96	1.35, 2.83	¡0.001	1.13	0.79, 1.63	0.5	0.97	0.66, 1.41	0.9
Jewish	2.01	1.13, 3.59	0.018	1.62	0.90, 2.92	0.11	0.73	0.35, 1.52	0.4
Other	2.13	1.41, 3.21	¡0.001	1.41	0.95, 2.10	0.091	0.81	0.52, 1.27	0.4

[1] OR = Odds Ratio, CI = Confidence Interval

Table S6. Weighted multinomial logistic regressions by type on plastic use (ref. Believers): Odds ratios, confidence intervals and p-values.

	Cynics			Doubters			Motivated		
**Characteristic**	**OR**	**95% CI**	**p-value**	**OR**	**95% CI**	**p-value**	**OR**	**95% CI**	**p-value**
Conspiracy Theory Score	1.27	1.10, 1.47	0.001	1.61	1.38, 1.89	¡0.001	1.24	1.02, 1.52	0.034
Environ. Knowledge Score	0.93	0.82, 1.05	0.3	0.74	0.64, 0.85	¡0.001	0.84	0.70, 1.00	0.053
Altruism Score	0.86	0.77, 0.96	0.007	0.79	0.71, 0.89	¡0.001	0.79	0.68, 0.92	0.003
Voted For									
Not Trump	—	—		—	—		—	—	
Trump	1.35	0.97, 1.86	0.072	1.72	1.21, 2.43	0.002	1.69	1.07, 2.67	0.025
Gender									
Not Woman	—	—		—	—		—	—	
Woman	0.52	0.41, 0.66	¡0.001	0.49	0.38, 0.64	¡0.001	0.66	0.48, 0.91	0.013
Subjective Income									
Better off	—	—		—	—		—	—	
The same	0.86	0.62, 1.20	0.4	1.33	0.90, 1.98	0.2	1.11	0.67, 1.84	0.7
Worse off	0.59	0.41, 0.85	0.004	1.00	0.66, 1.52	¿0.9	1.20	0.71, 2.01	0.5
Education									
No College	—	—		—	—		—	—	
College	0.68	0.53, 0.88	0.003	0.82	0.62, 1.07	0.14	1.43	1.03, 2.00	0.035
Age									
65 or older	—	—		—	—		—	—	
45 to 64	1.44	1.06, 1.96	0.019	2.07	1.48, 2.90	¡0.001	1.57	1.04, 2.40	0.034
30 to 44	2.05	1.43, 2.94	¡0.001	2.61	1.75, 3.91	¡0.001	1.73	1.02, 2.90	0.040
18 to 29	2.02	1.35, 3.01	¡0.001	3.26	2.10, 5.07	¡0.001	2.28	1.32, 3.94	0.003
Political ID	1.10	1.00, 1.20	0.040	1.14	1.04, 1.26	0.008	1.04	0.92, 1.19	0.5
Religion									
No religion	—	—		—	—		—	—	
Protestant	1.23	0.88, 1.72	0.2	1.10	0.76, 1.57	0.6	0.75	0.48, 1.18	0.2
Catholic	1.23	0.87, 1.73	0.2	1.26	0.88, 1.82	0.2	0.84	0.53, 1.32	0.4
Jewish	2.11	1.23, 3.63	0.007	0.99	0.51, 1.94	¿0.9	0.85	0.39, 1.88	0.7
Other	1.31	0.90, 1.91	0.2	0.80	0.53, 1.23	0.3	0.68	0.39, 1.16	0.2

[1] OR = Odds Ratio, CI = Confidence Interval

Table S7. Weighted multinomial logistic regressions by type on water use (ref. Believers): Odds ratios, confidence intervals and p-values.

	**Cynics**			**Doubters**			**Motivated**		
**Characteristic**	**OR**	**95% CI**	**p-value**	**OR**	**95% CI**	**p-value**	**OR**	**95% CI**	**p-value**
Conspiracy Theory Score	1.26	1.09, 1.45	0.002	1.37	1.18, 1.59	¡0.001	1.42	1.13, 1.78	0.002
Environ. Knowledge Score	0.93	0.82, 1.05	0.2	0.83	0.72, 0.94	0.005	0.71	0.57, 0.89	0.002
Altruism Score	0.84	0.75, 0.93	¡0.001	0.76	0.68, 0.85	¡0.001	0.74	0.62, 0.87	¡0.001
Voted For									
Not Trump	—	—		—	—		—	—	
Trump	1.09	0.78, 1.51	0.6	1.89	1.35, 2.67	¡0.001	2.81	1.69, 4.67	¡0.001
Gender									
Not Woman	—	—		—	—		—	—	
Woman	0.87	0.69, 1.09	0.2	0.66	0.52, 0.84	¡0.001	0.76	0.53, 1.10	0.15
Subjective Income									
Better off	—	—		—	—		—	—	
The same	0.85	0.61, 1.19	0.3	1.50	1.01, 2.22	0.042	1.28	0.73, 2.25	0.4
Worse off	0.63	0.44, 0.89	0.009	1.23	0.82, 1.85	0.3	1.05	0.58, 1.89	0.9
Education									
No College	—	—		—	—		—	—	
College	0.87	0.68, 1.11	0.3	0.92	0.71, 1.19	0.5	1.55	1.06, 2.27	0.023
Age									
65 or older	—	—		—	—		—	—	
45 to 64	1.15	0.85, 1.54	0.4	1.69	1.25, 2.30	¡0.001	2.00	1.21, 3.31	0.007
30 to 44	2.07	1.46, 2.94	¡0.001	2.36	1.61, 3.47	¡0.001	3.13	1.72, 5.68	¡0.001
18 to 29	2.51	1.69, 3.73	¡0.001	3.11	2.02, 4.79	¡0.001	4.30	2.26, 8.16	¡0.001
Political ID	1.06	0.97, 1.16	0.2	1.18	1.08, 1.30	¡0.001	1.08	0.94, 1.25	0.3
Religion									
No religion	—	—		—	—		—	—	
Protestant	1.25	0.90, 1.73	0.2	0.93	0.66, 1.31	0.7	0.57	0.34, 0.97	0.036
Catholic	1.20	0.86, 1.69	0.3	1.17	0.83, 1.66	0.4	0.87	0.52, 1.44	0.6
Jewish	3.18	1.81, 5.59	¡0.001	1.23	0.62, 2.44	0.6	1.04	0.41, 2.62	¿0.9
Other	1.20	0.83, 1.73	0.3	0.72	0.48, 1.08	0.11	0.60	0.33, 1.09	0.10

[1] OR = Odds Ratio, CI = Confidence Interval

Table S8. Multinomial logistic regressions by type on composting (ref. Believers): Odds ratios, confidence intervals and p-values.

	Cynics			Doubters			Motivated		
**Characteristic**	**OR**	**95% CI**	**p-value**	**OR**	**95% CI**	**p-value**	**OR**	**95% CI**	**p-value**
Conspiracy Theory Score	0.88	0.74, 1.04	0.12	0.92	0.78, 1.09	0.3	1.30	1.02, 1.65	0.031
Environ. Knowledge Score	1.04	0.91, 1.20	0.6	0.87	0.75, 1.00	0.055	0.79	0.63, 0.99	0.039
Altruism Score	0.82	0.72, 0.92	0.001	0.73	0.65, 0.83	¡0.001	0.81	0.67, 0.96	0.018
Voted For									
Not Trump	—	—		—	—		—	—	
Trump	1.01	0.69, 1.50	¿0.9	2.27	1.55, 3.32	¡0.001	2.27	1.30, 3.95	0.004
Gender									
Not Woman	—	—		—	—		—	—	
Woman	1.07	0.81, 1.41	0.7	0.68	0.52, 0.89	0.005	0.83	0.56, 1.23	0.3
Subjective Income									
Better off	—	—		—	—		—	—	
The same	1.00	0.68, 1.46	¿0.9	1.37	0.92, 2.05	0.12	1.81	0.96, 3.39	0.065
Worse off	0.76	0.51, 1.15	0.2	1.56	1.03, 2.37	0.037	1.43	0.74, 2.76	0.3
Education									
No College	—	—		—	—		—	—	
College	0.84	0.63, 1.12	0.2	0.70	0.53, 0.93	0.013	1.20	0.80, 1.81	0.4
Age									
65 or older	—	—		—	—		—	—	
45 to 64	1.08	0.76, 1.55	0.7	1.42	1.01, 2.00	0.046	2.68	1.58, 4.56	¡0.001
30 to 44	1.26	0.83, 1.91	0.3	1.05	0.70, 1.59	0.8	1.77	0.93, 3.37	0.080
18 to 29	1.31	0.83, 2.07	0.3	1.27	0.80, 2.01	0.3	1.87	0.91, 3.82	0.087
Political ID	0.99	0.89, 1.10	0.9	1.04	0.94, 1.15	0.5	0.98	0.84, 1.15	0.8
Religion									
No religion	—	—		—	—		—	—	
Protestant	1.91	1.28, 2.84	0.001	1.39	0.94, 2.05	0.10	1.15	0.67, 1.98	0.6
Catholic	1.23	0.83, 1.82	0.3	1.18	0.81, 1.73	0.4	0.46	0.25, 0.85	0.014
Jewish	2.06	1.10, 3.86	0.023	0.78	0.40, 1.55	0.5	0.60	0.21, 1.70	0.3
Other	1.35	0.88, 2.06	0.2	0.71	0.46, 1.09	0.11	0.76	0.42, 1.38	0.4

[1] OR = Odds Ratio, CI = Confidence Interval

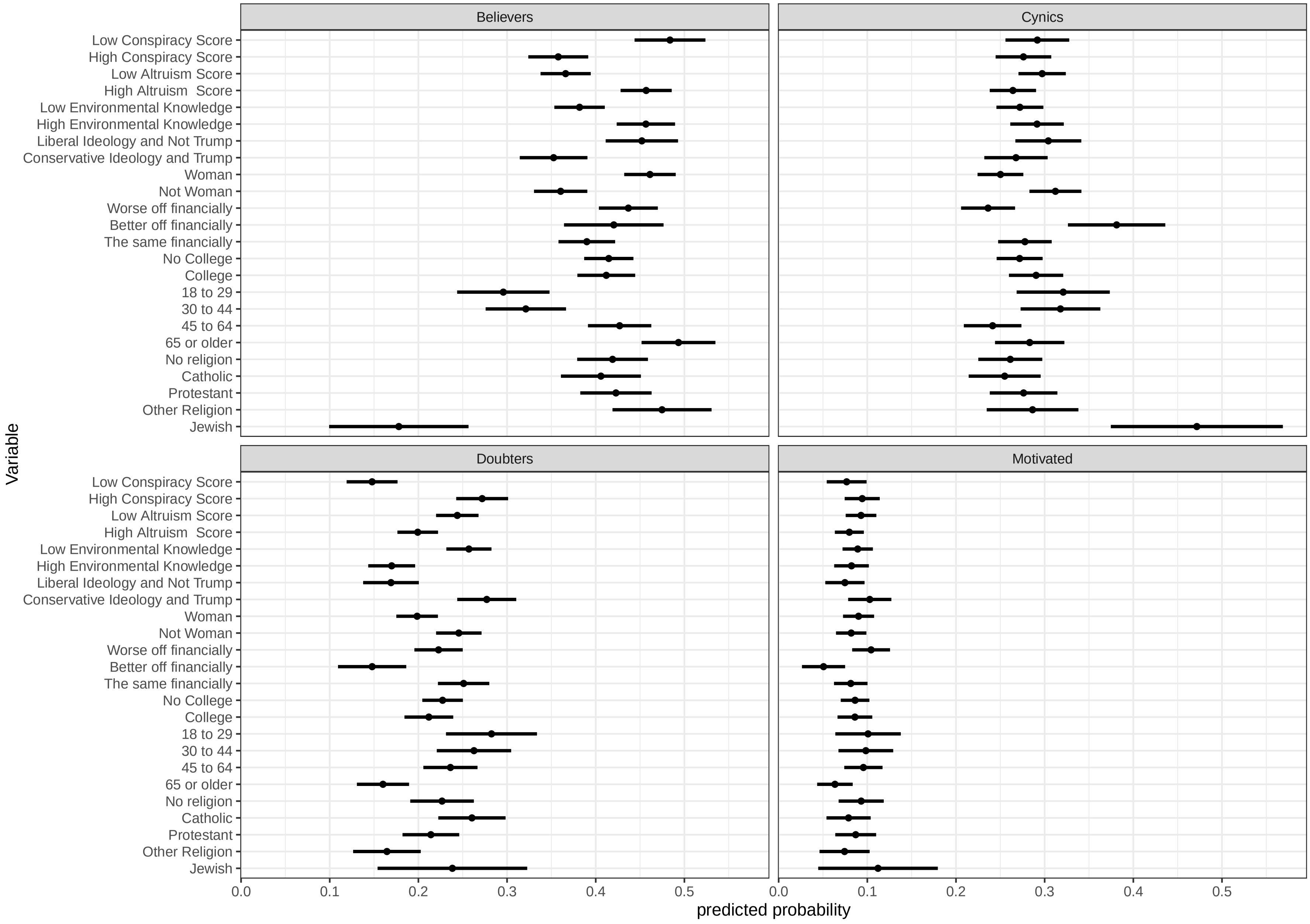

Figure S3. Predicted probability of being a believer, cynic, doubter or motivated on use of electricity

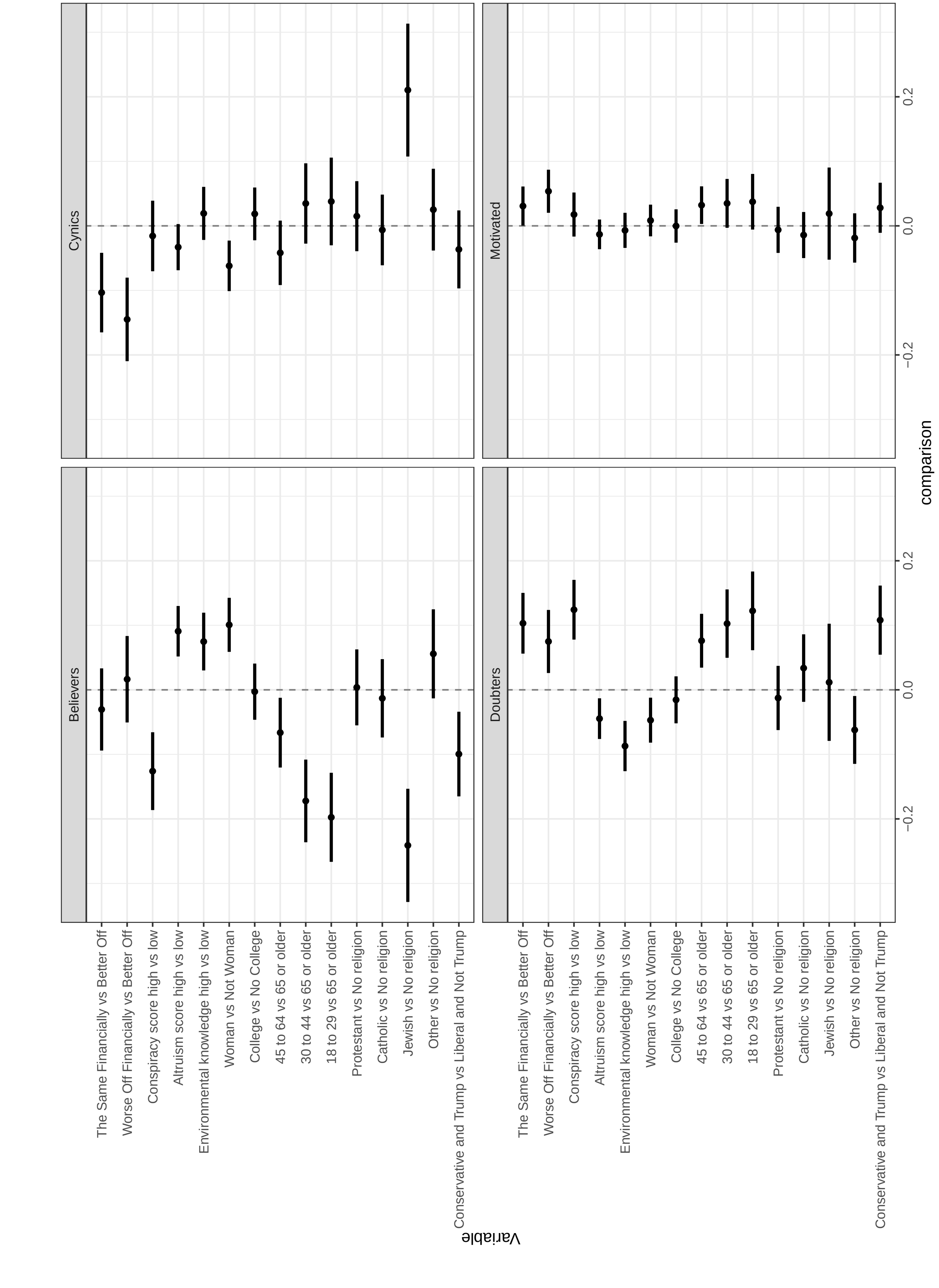

Figure S4. Change in probability of being believers, motivated, doubters, cynics for electricity use

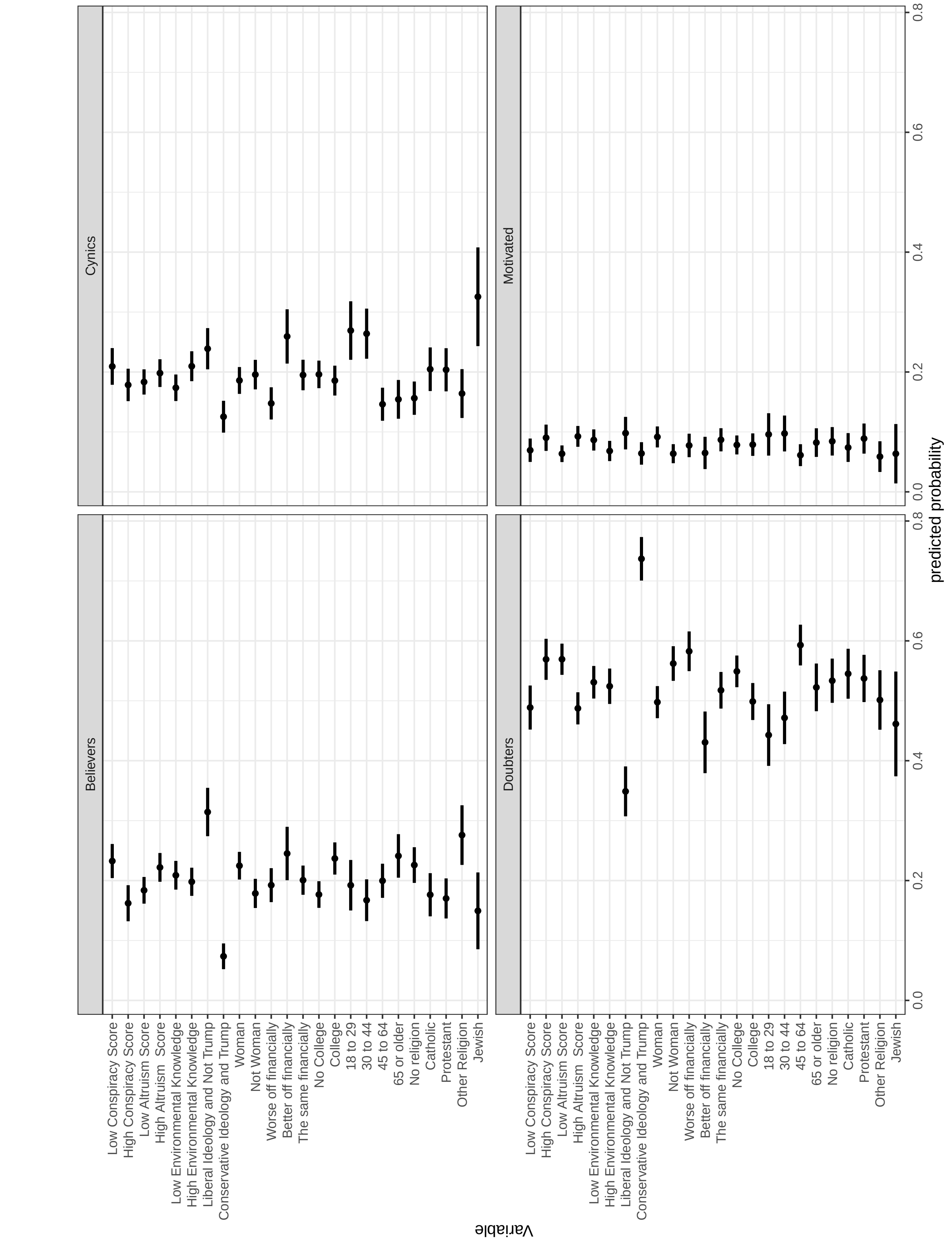

Figure S5. Predicted probability of being a believer, cynic, doubter or motivated on meat consumption

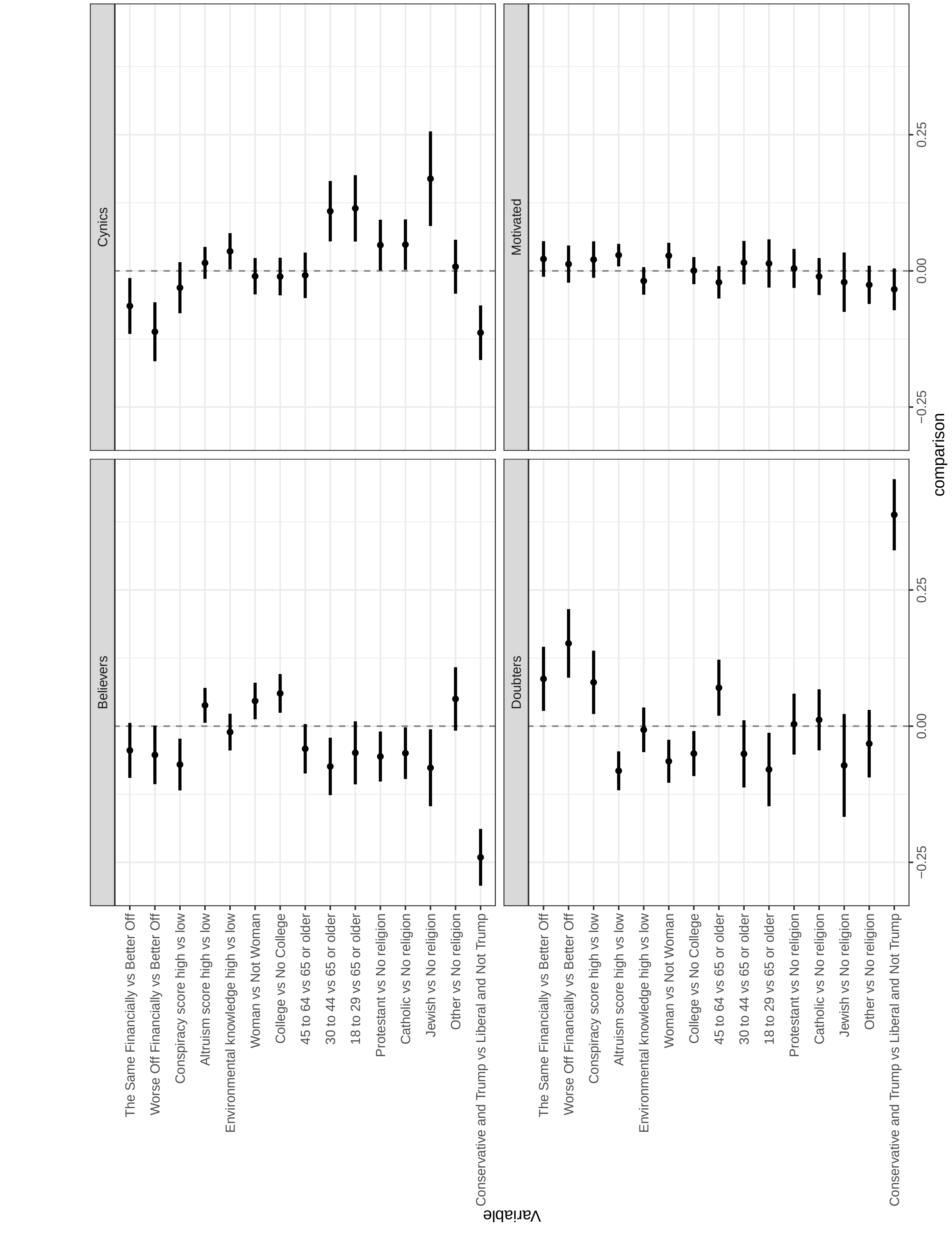

Figure S6. Change in probability of being believers, motivated, doubters, cynics for meat consumption

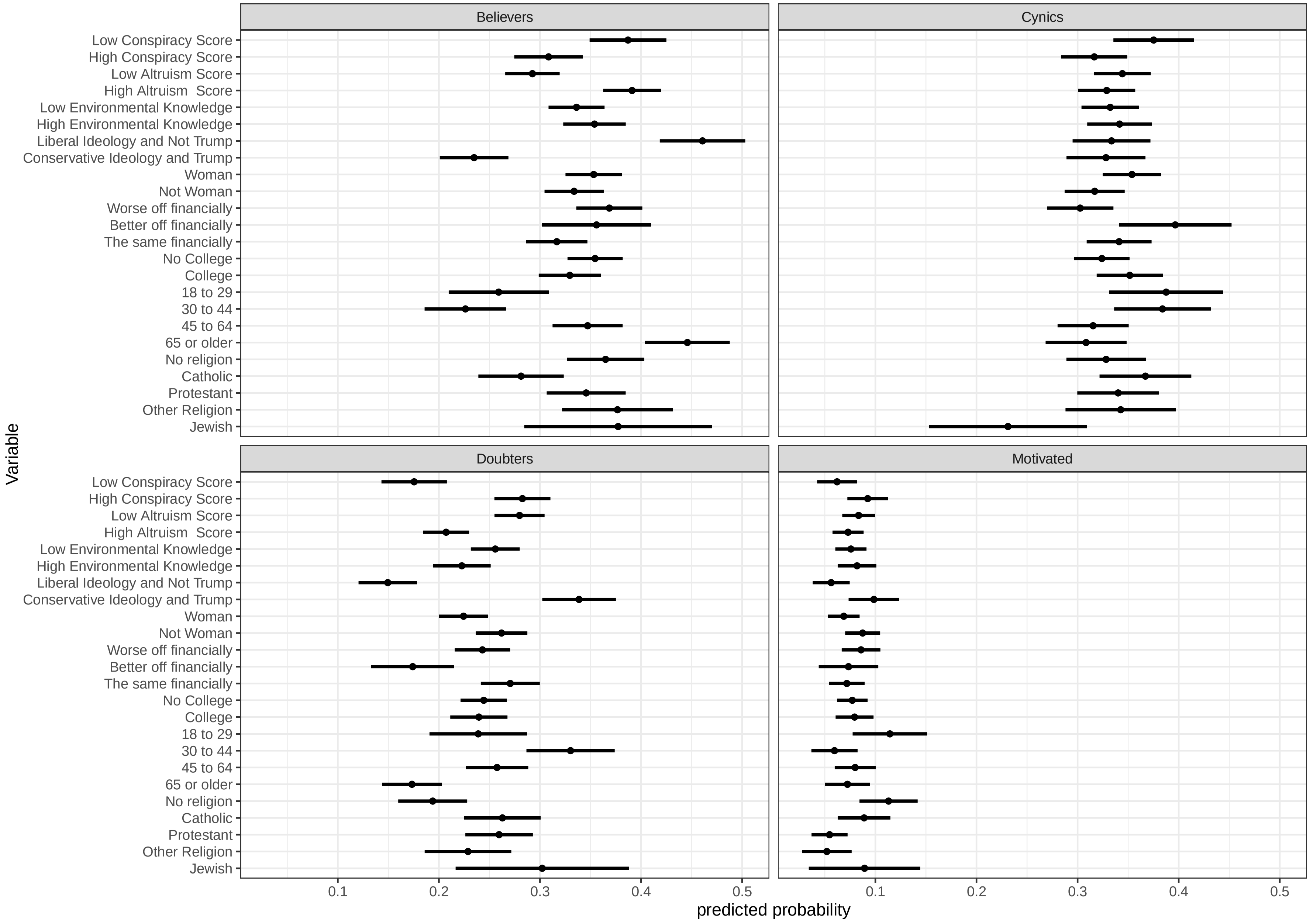

Figure S7. Predicted probability of being a believer, cynic, doubter or motivated on driving behavior

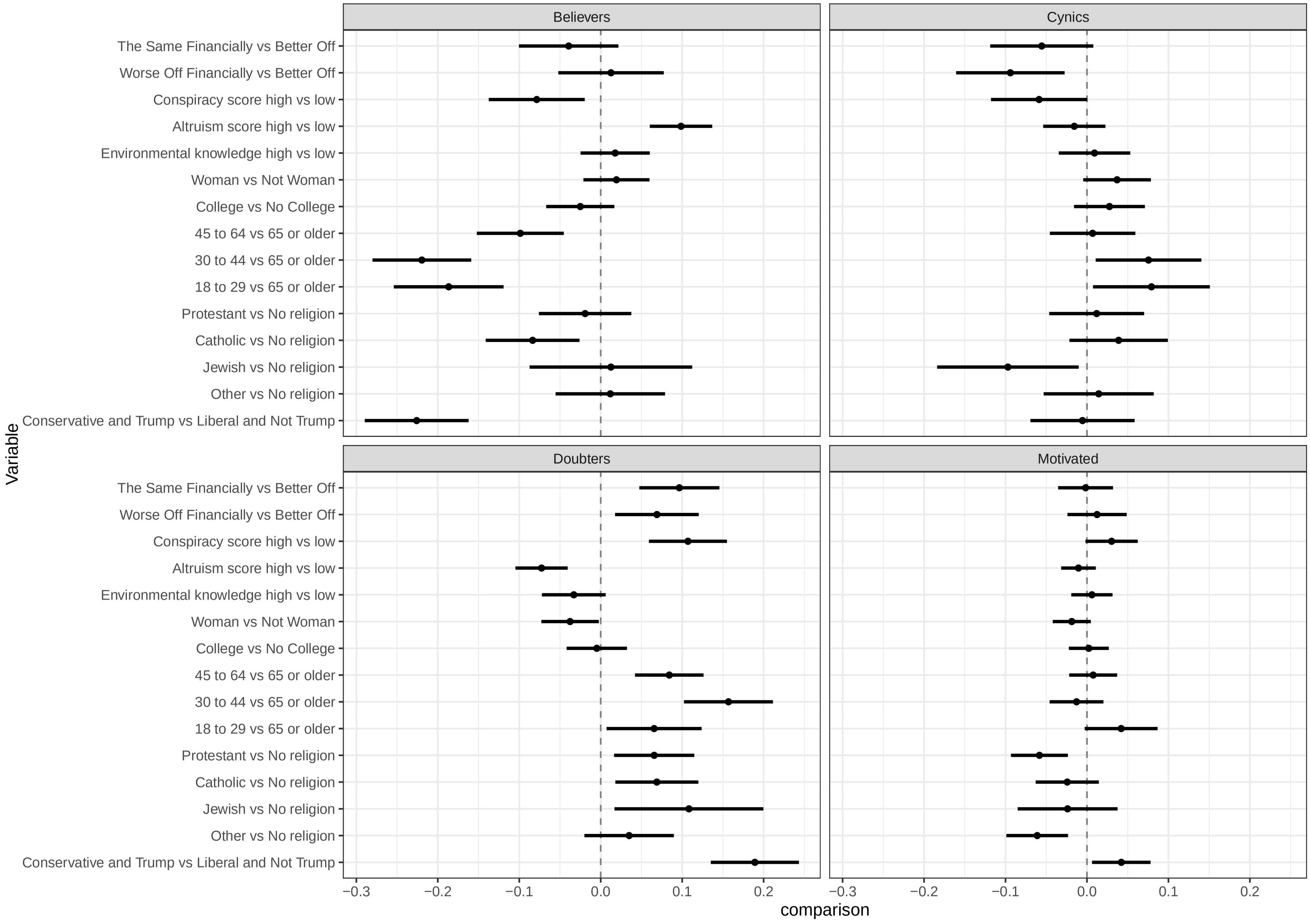

Figure S8. Change in probability of being believers, motivated, doubters, cynics for driving behavior

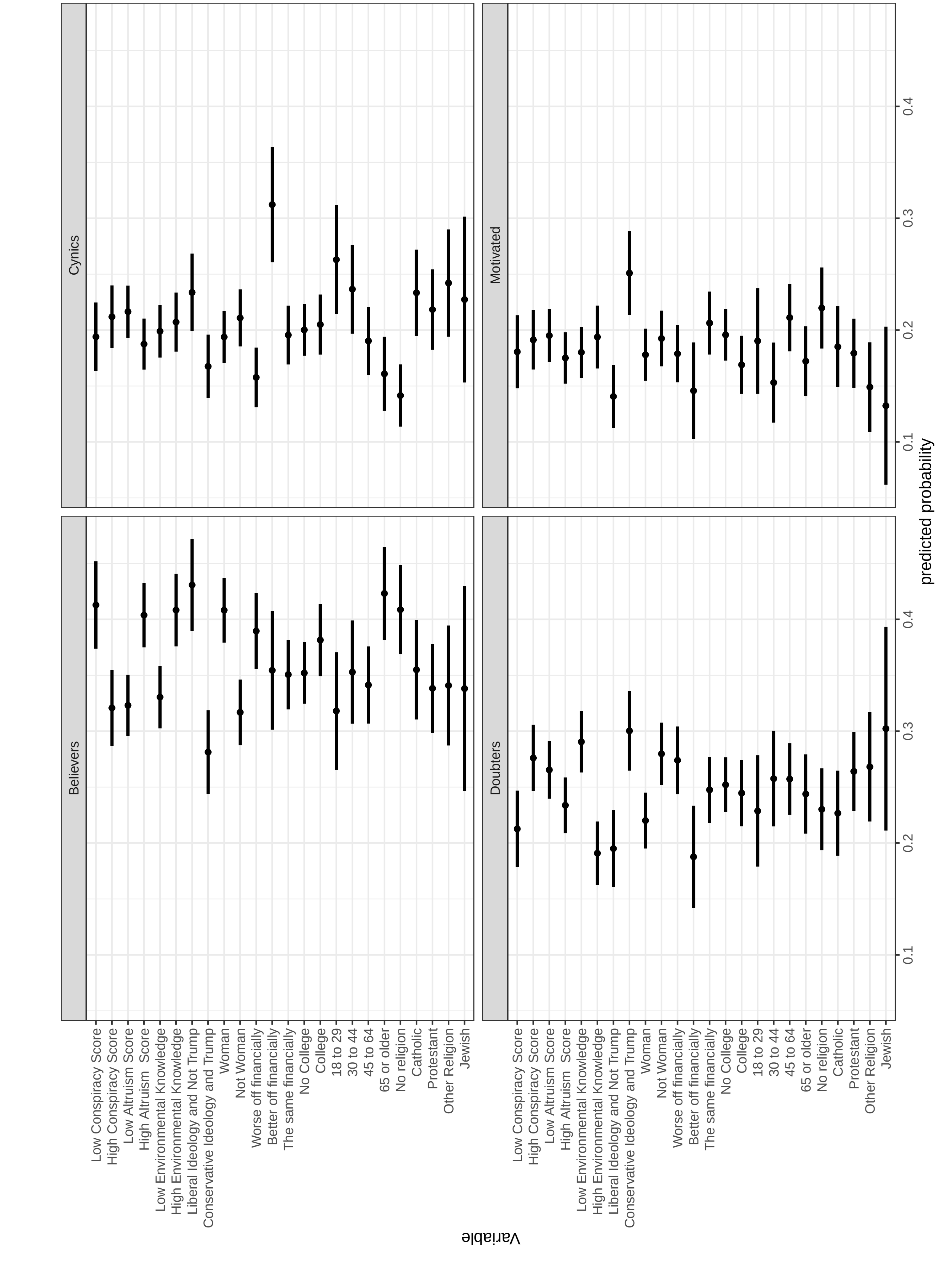

Figure S9. Predicted probability of being a believer, cynic, doubter or motivated on food waste

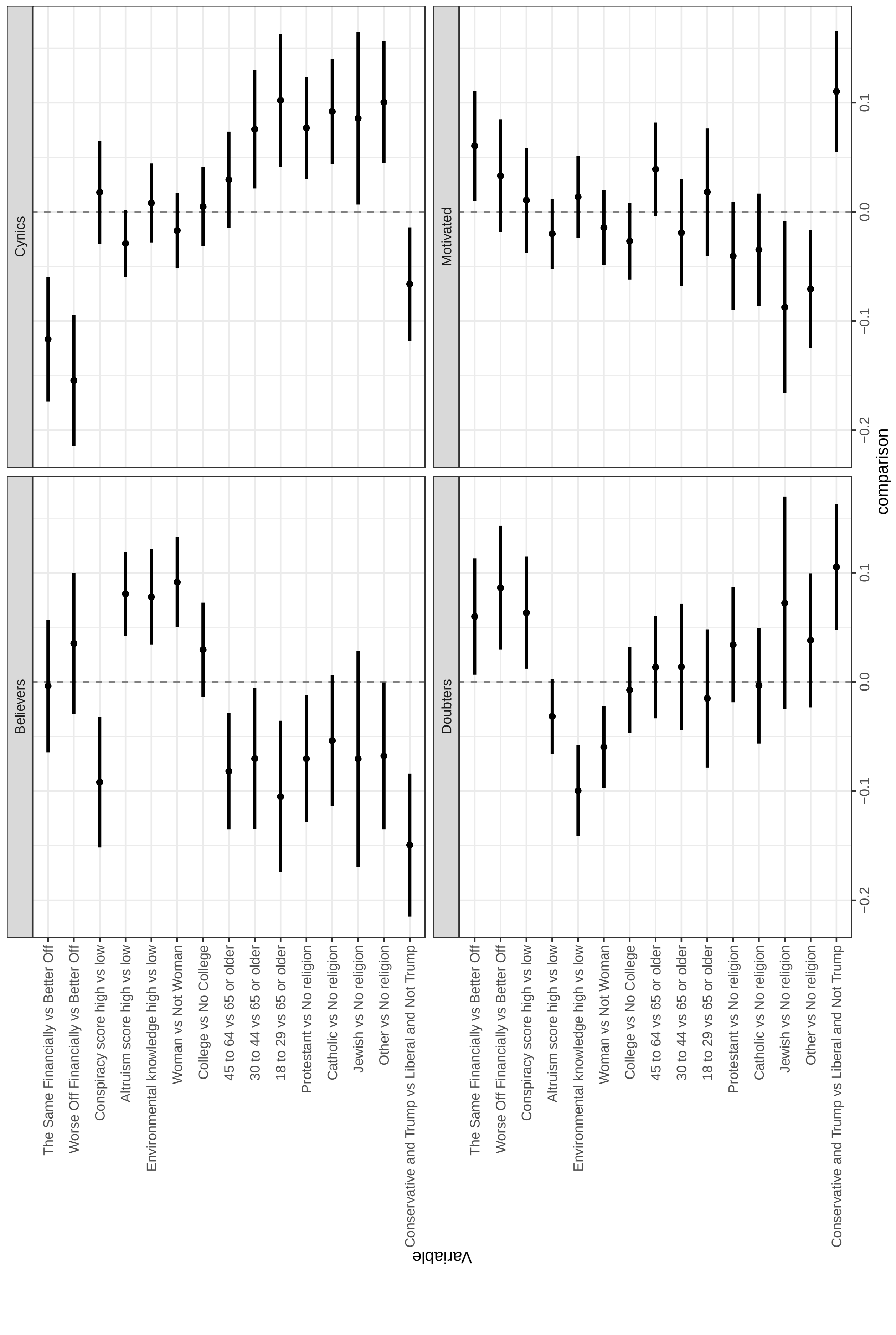

Figure S10. Change in probability of being believers, motivated, doubters, cynics for food waste

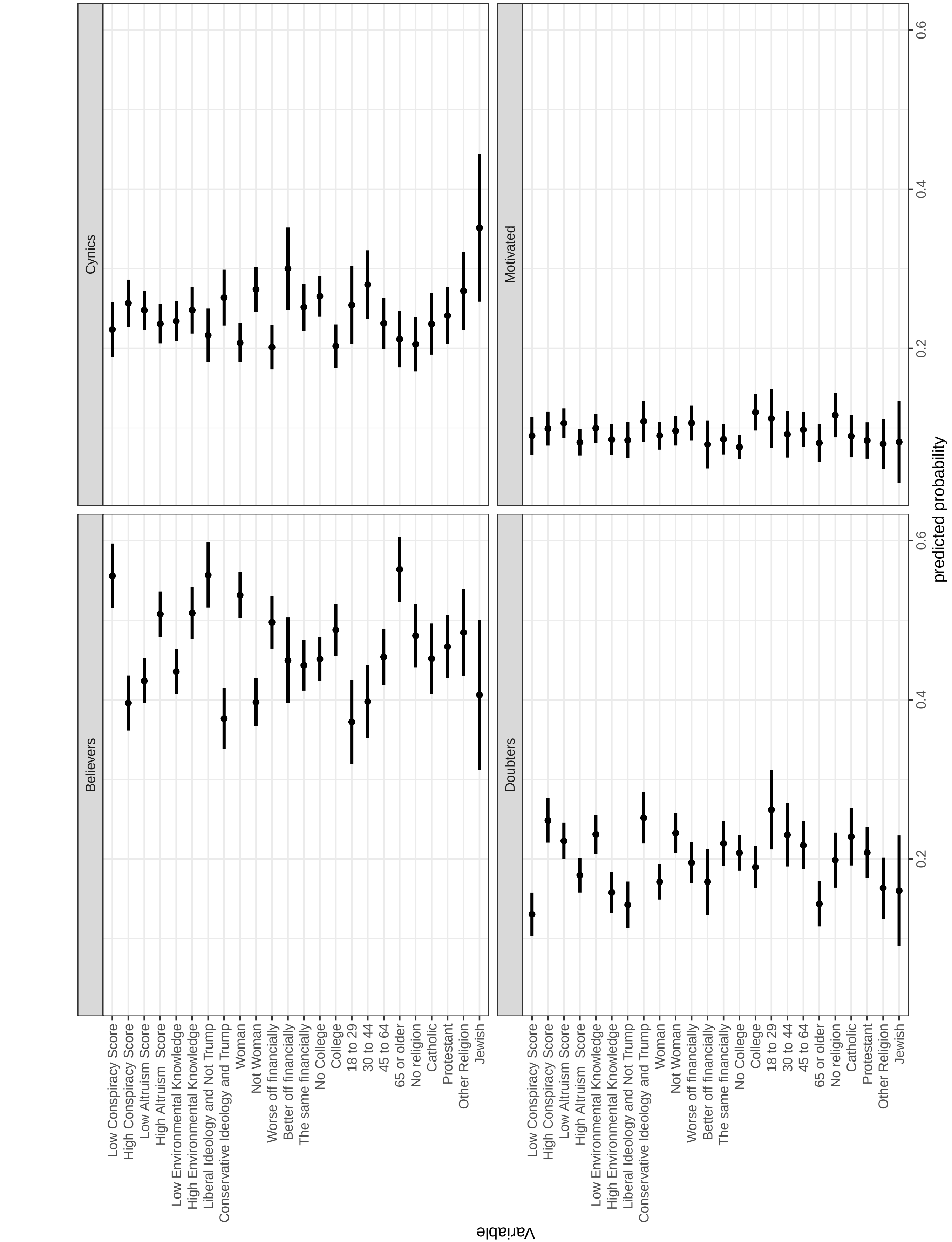

Figure S11. Predicted probability of being a believer, cynic, doubter or motivated on plastic use

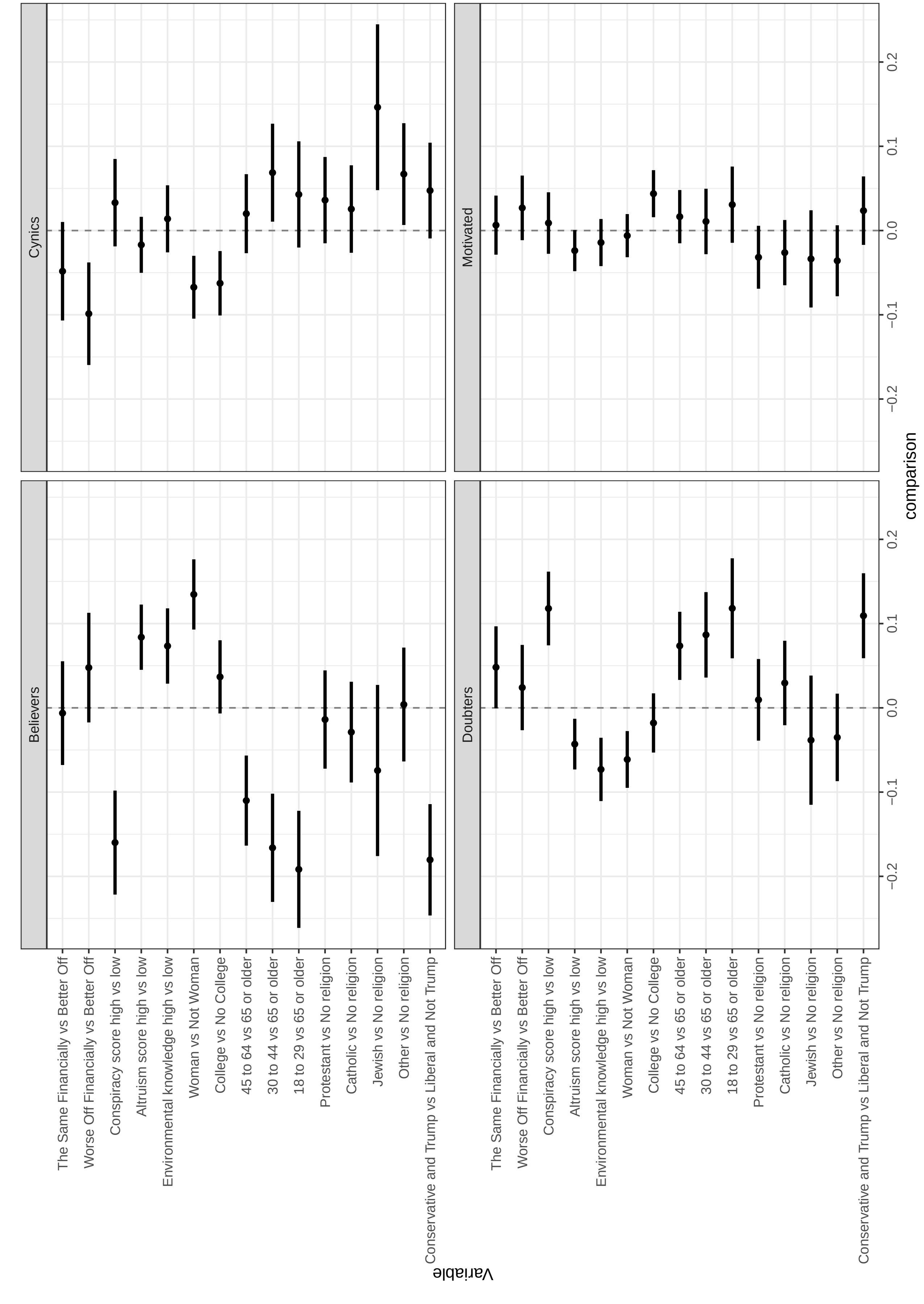

Figure S12. Change in probability of being believers, motivated, doubters, cynics for plastic use

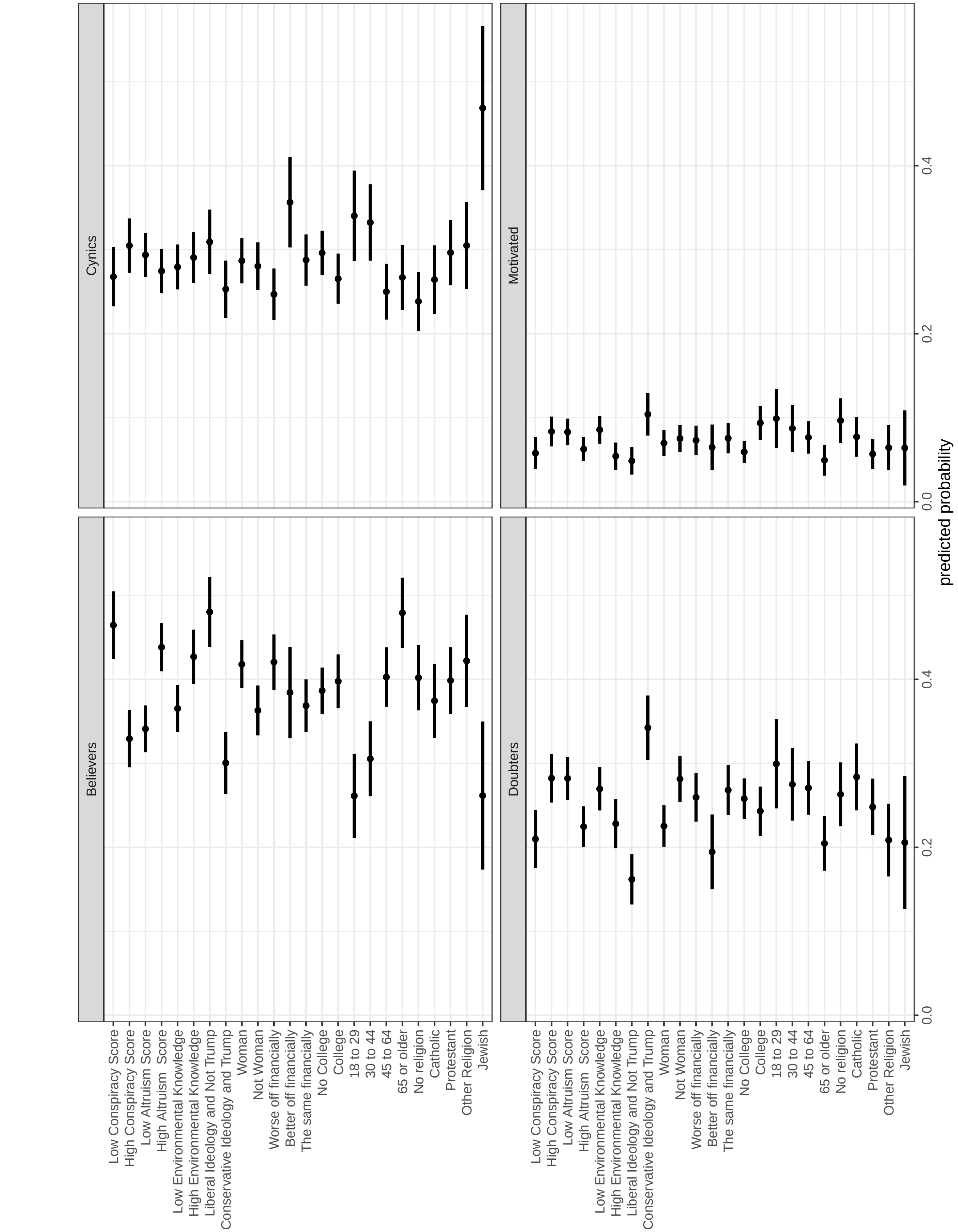

Figure S13. Predicted probability of being a believer, cynic, doubter or motivated on water use

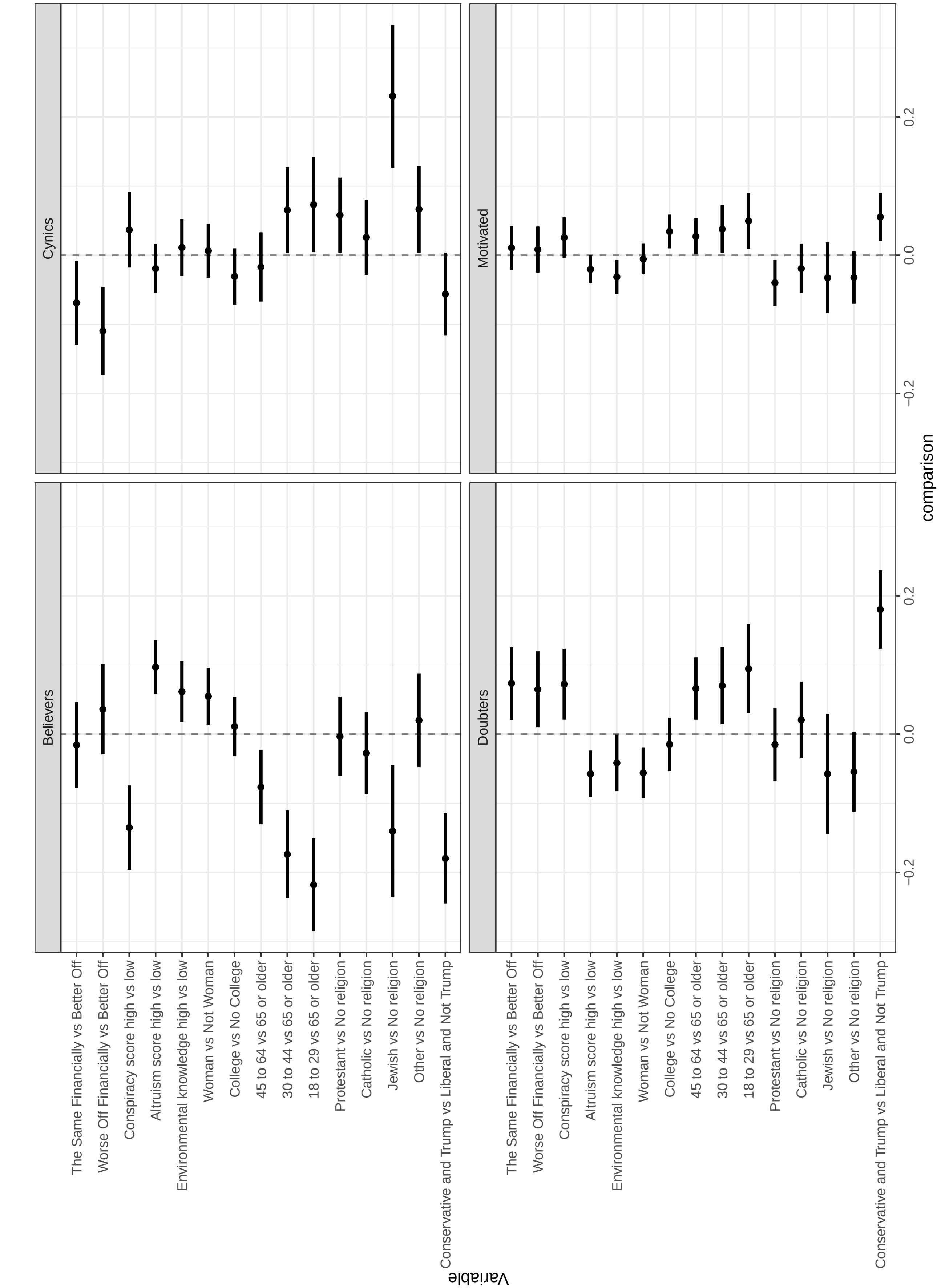

Figure S14. Change in probability of being believers, motivated, doubters, cynics for water use

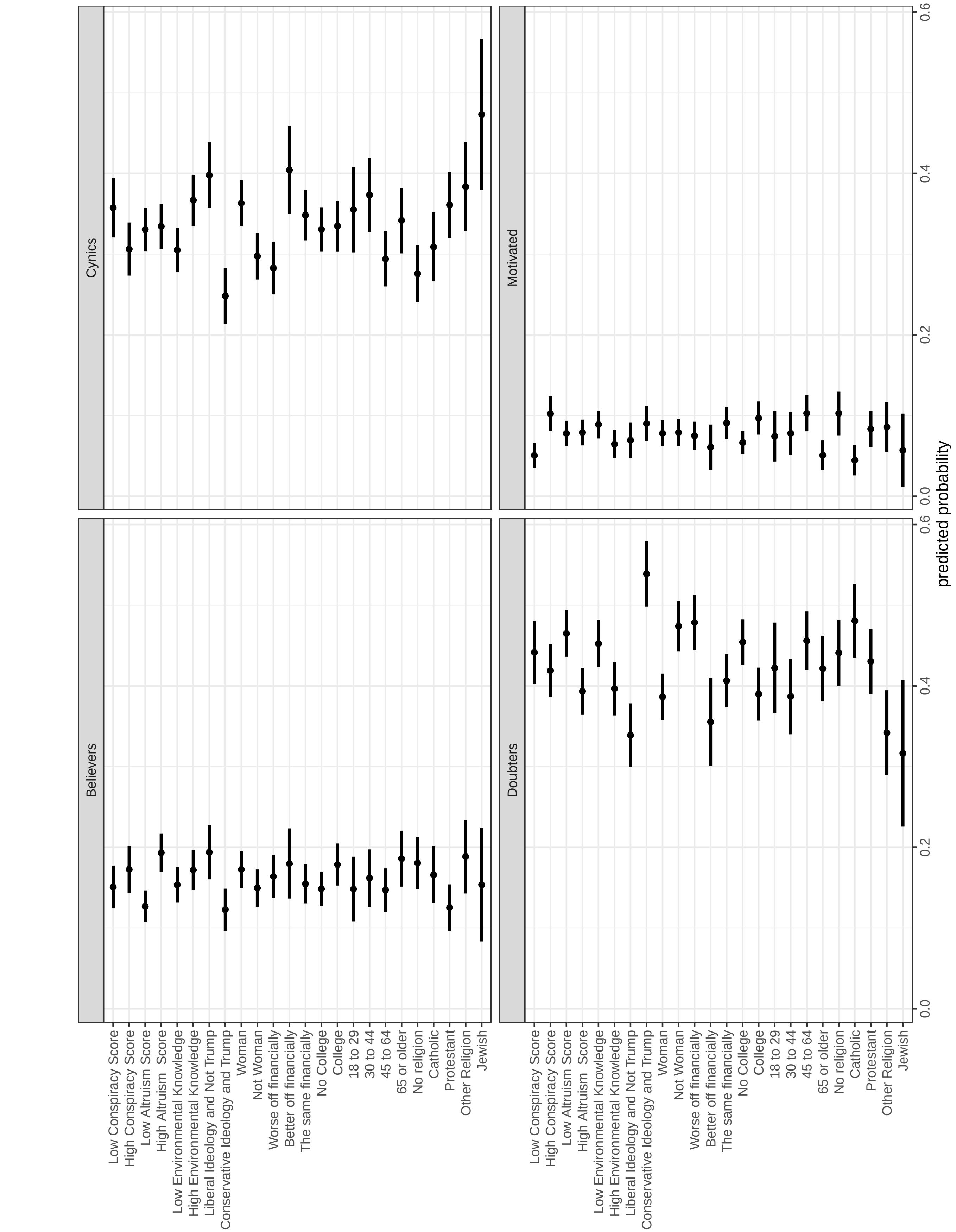

Figure S15. Predicted probability of being a believer, cynic, doubter or motivated on composting

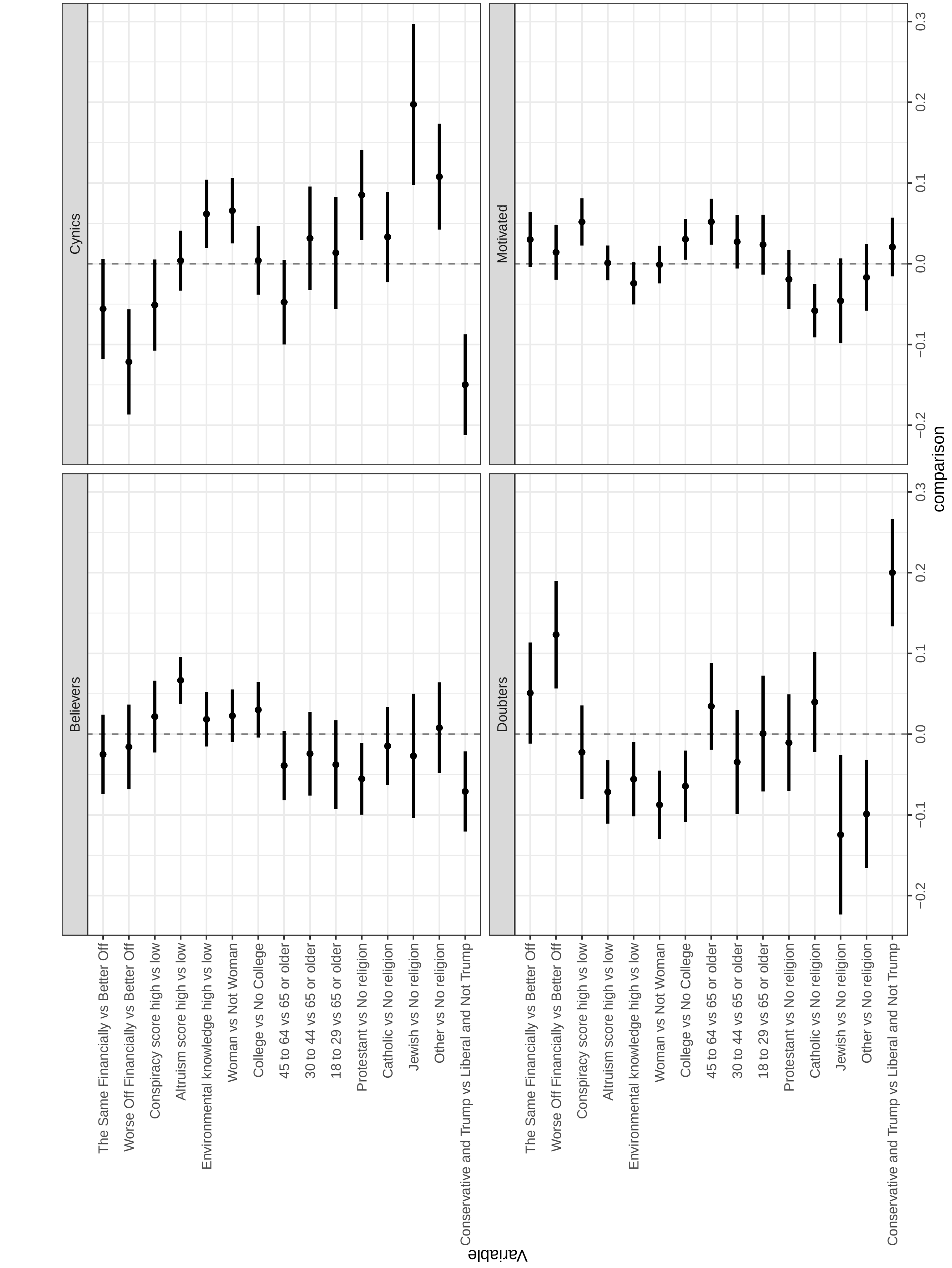

Figure S16. Change in probability of being believers, motivated, doubters, cynics for composting

3. Robustness Checks

In this section, as a robustness check, we conducted the main analyses excluding "not sure" responses, since we recognize the potential for ambiguity in their placement within categories. The results of these analyses, presented in the section below, indicate that the overall findings remain consistent even when these responses are excluded.

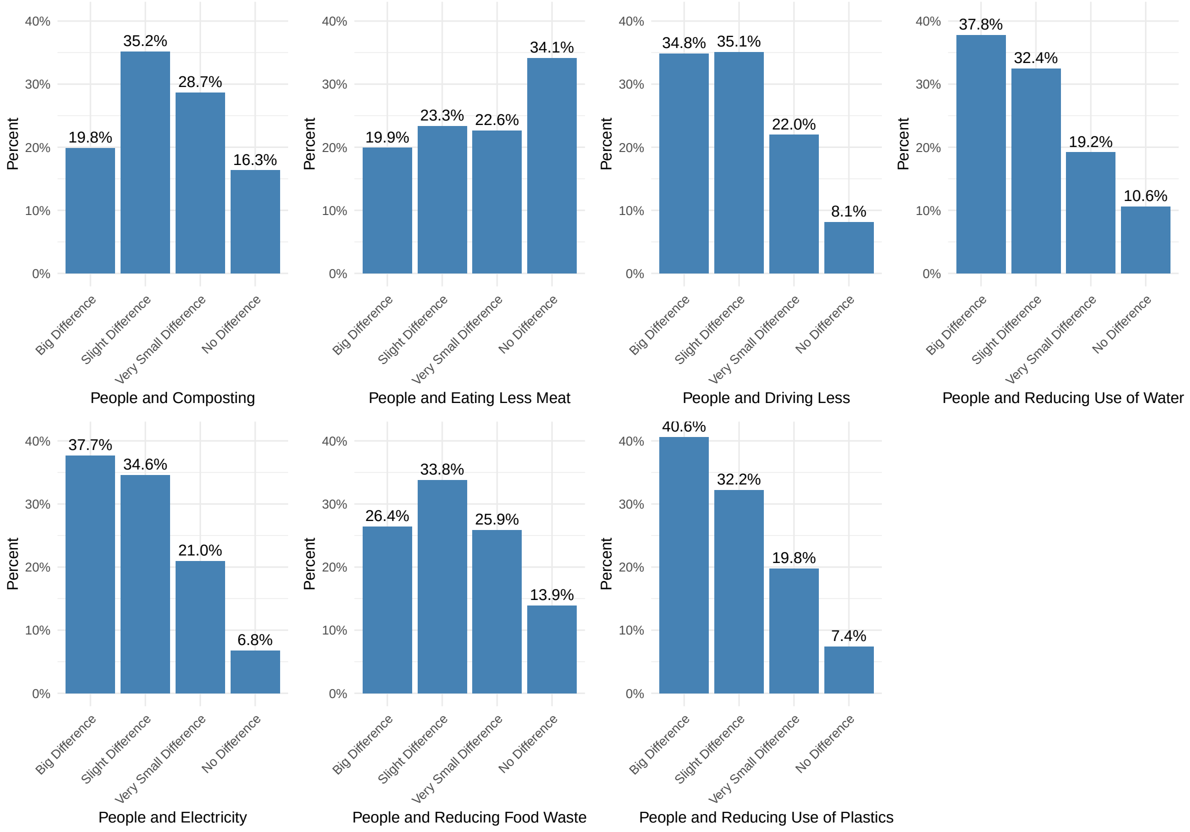

Figure S17. Percentage of people who think each sustainable behavior makes or does not make a difference (excluding not sure)

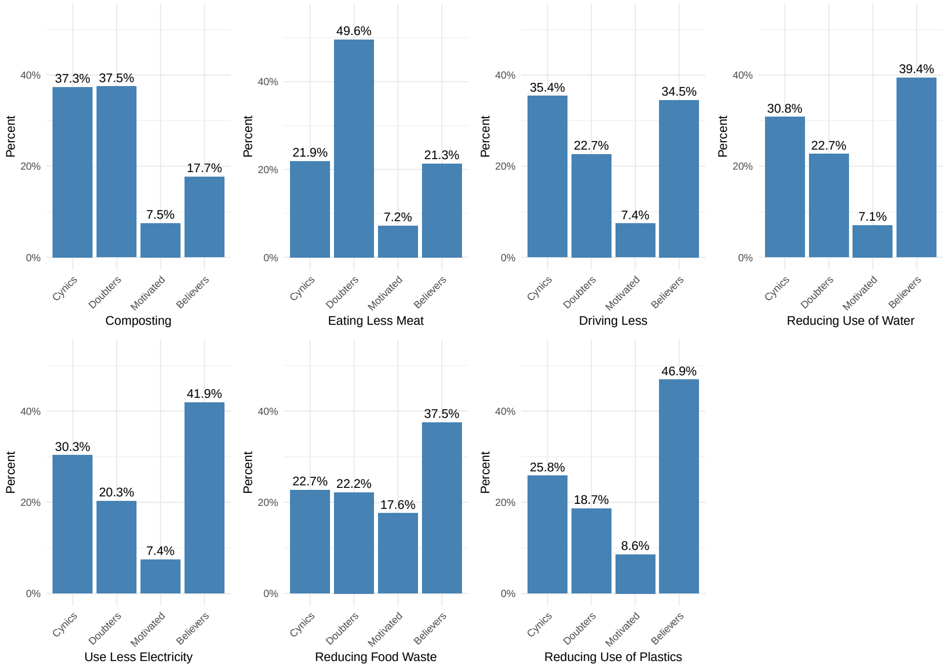

Figure S18. Percentage of people by category (cynics, doubters, motivated and believers) for each sustainable behavior (excluding not sure)

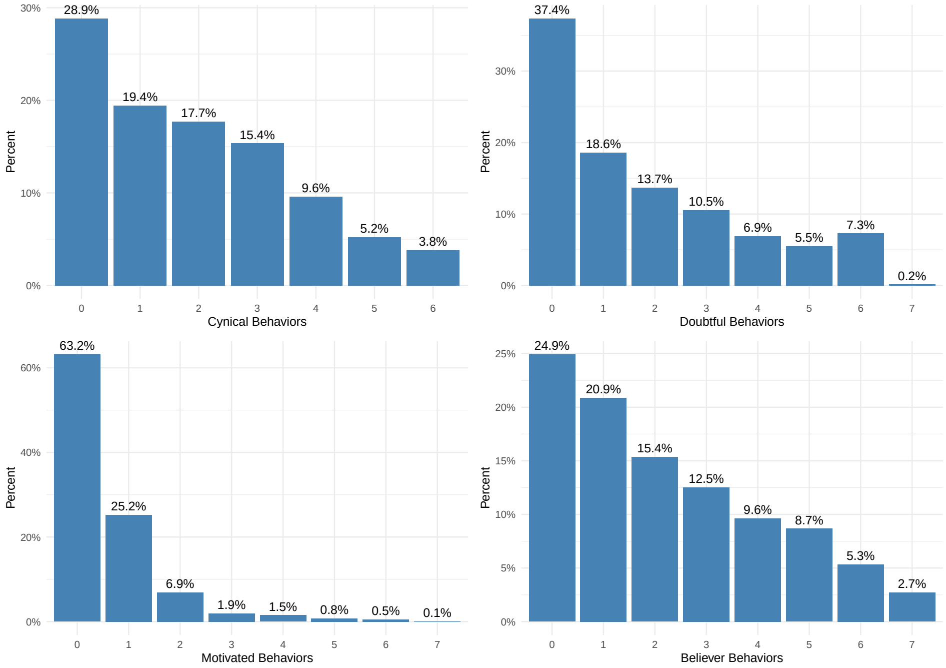

Figure S19. Percentage of people that engage in n (0 to 7) cynical, doubtful, motivated, or believer behaviors (excluding not sure)

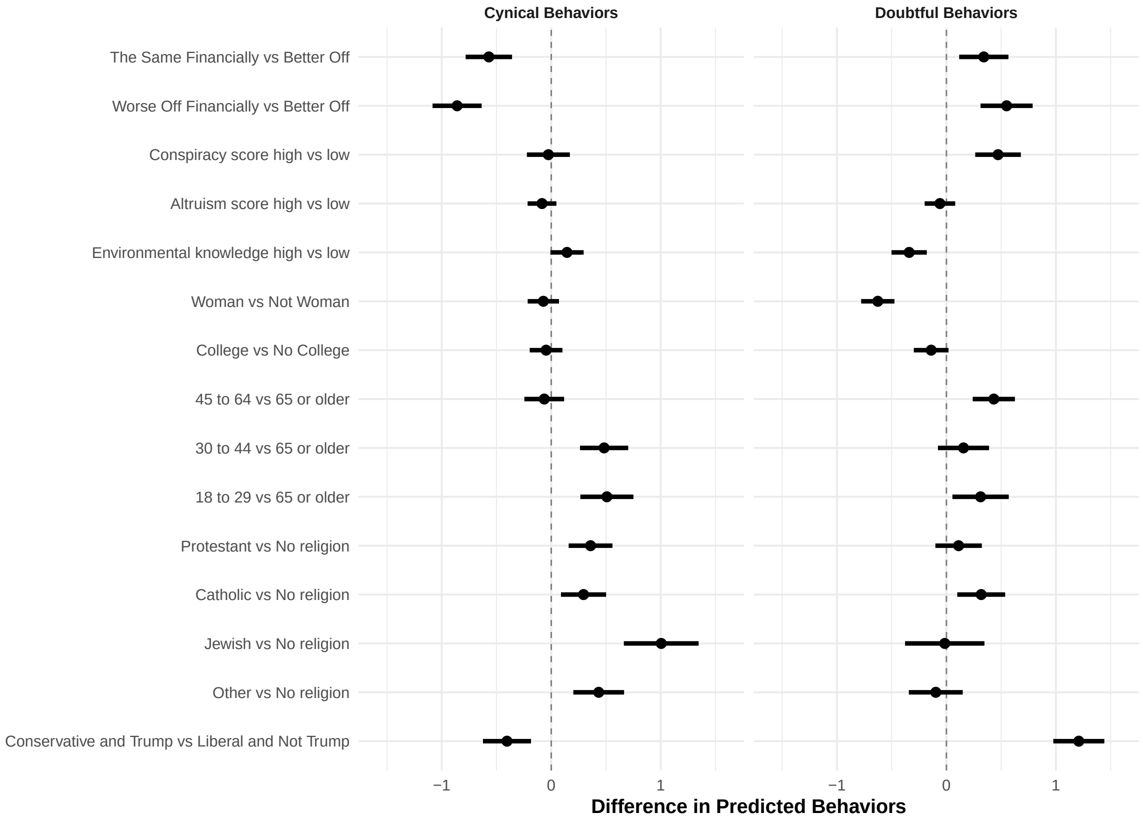

Figure S20. Differences in Predicted Engagement in Cynical and Doubtful Behaviors Across Different Quantities of Interest (excluding not sure)

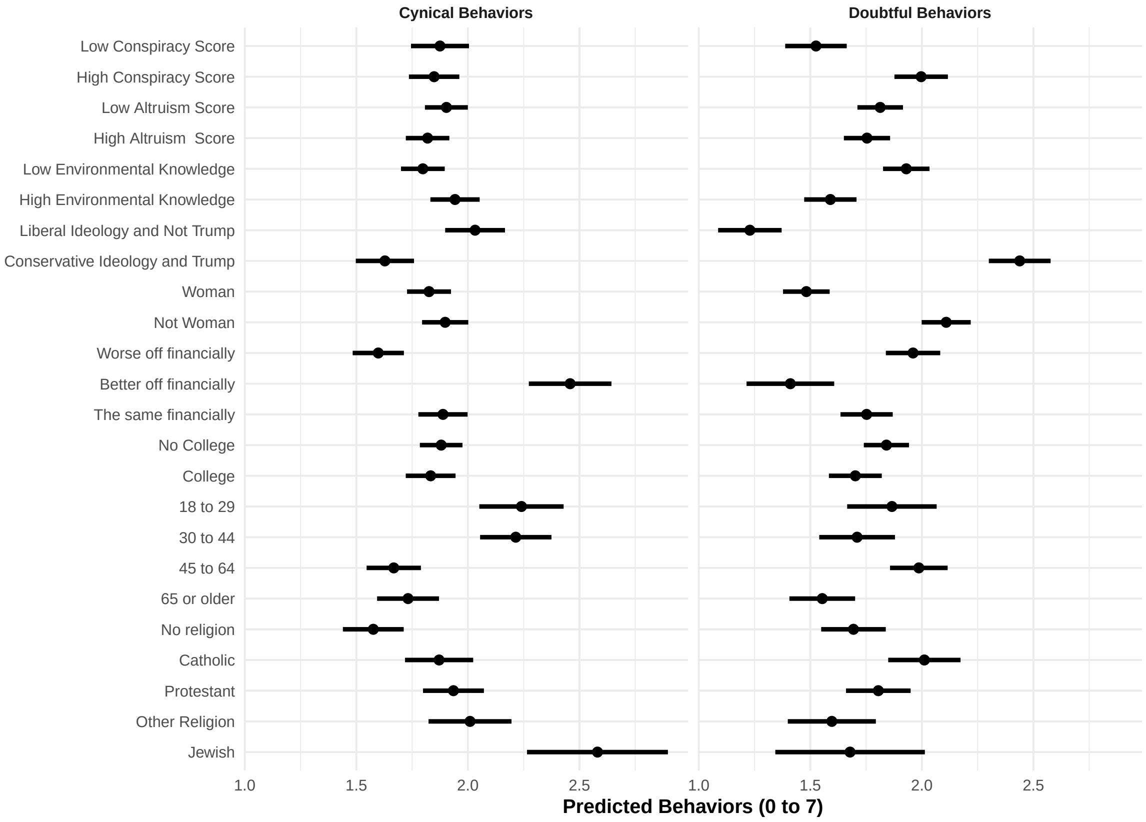

Figure S21. Predicted Engagement in Cynical and Doubtful Behaviors Across Different Quantities of Interest (excluding not sure)
